# Supplementary material for: Fixation patterns in simple choice reflect optimal information sampling
Source: PLoS Comput Biol. 2021 Mar 26;17(3):e1008863. doi: 10.1371/journal.pcbi.1008863 (PMC8026028; doi:10.1371/journal.pcbi.1008863)
Supplement: S1 Appendix — Includes descriptions of the tasks for the datasets we model, individual fitting methods and summary of results, parameter recovery results, aDDM implementation and validation, derivations for the value of information features, and a validation of the policy approximation method. (PDF) [file pcbi.1008863.s001.pdf]

Supporting Information

Binary choice: Task description

This data was initially reported by Krajbich et al. in 2010. For the convenience of the reader, we include the task description from the original paper [1]. The experiment consisted of 39 Caltech students. Only subjects who self-reported regularly eating the snack foods (for example, potato chips and candy bars) used in the experiment and not being on a diet were allowed to participate. These steps were taken to ensure that the food items we used would be motivationally relevant. This would not have been the case if the subjects did not like junk food. Subjects were asked to refrain from eating for 3 h before the start of the experiment. After the experiment they were required to stay in the room with the experimenter for 30 min while eating the food item that they chose in a randomly selected trial (see below). Subjects were not allowed to eat anything else during this time.

In an initial rating phase subjects entered liking ratings for 70 different foods using an on-screen slider bar (how much would you like to eat this at the end of the experiment?, scale -10 to 10). The initial location of the slider was randomized to reduce anchoring effects. This rating screen had a free response time. The food was kept in the room with the subjects during the experimental session to assure them that all the items were available. Furthermore, subjects briefly saw all the items at this point so that they could effectively use the rating scale.

In the choice phase, subjects made their choices by pressing the left or right arrow keys on the keyboard. The choice screen had a free response time. Food items that received a negative rating in the rating phase of the experiment were excluded from the choice phase. The items shown in each trial were chosen pseudo-randomly according to the following rules: (i) no item was used in more than 6 trials; (ii) the difference in liking ratings between the two items was constrained to be 5 or less; (iii) if at some point in the experiment (i) and (ii) could no longer both be satisfied, then the difference in allowable liking ratings was expanded to 7, but these trials occurred for only 5 subjects and so were discarded from the analyses. The spatial location of the items was randomized. After subjects indicated their choice, a yellow box was drawn around the chosen item (with the other item still on-screen) and displayed for 1 s, followed by a fixation screen before the beginning of the next trial.

Subjects fixation patterns were recorded at 50 Hz using a Tobii desktop-mounted eye-tracker. Before each choice trial, subjects were required to maintain a fixation at the center of the screen for 2 s before the items would appear, ensuring that subjects began every choice fixating on the same location.

Trinary choice: Task description

This data was initially reported by Krajbich and Rangel in 2011. For the convenience of the reader, we include the task description from the original paper [2].

Thirty Caltech students participated in the experiment. The screening, pre-experimental instructions, eye-tracking and liking rating phase were identical to those used in the binary choice task described in the previous section.

In the choice phase, subjects made their choices using the keyboard. The choice screen had a free response time. The items shown in each trial were randomly chosen. In all trials the three items were displayed in a triangular formation with the left and right items at the same vertical position, and the center item at the opposite vertical position. In half of the trials the center item was on the top half of the screen, and in the other half it was on the bottom half of the screen. Subjects indicated their choice by pressing the left, down, or right arrow keys for the left, center, and right items, respectively. After subjects indicated their choice, a yellow box was drawn around the chosen item (with the other item still on the screen) and displayed for 1 s, followed by a fixation screen, before the beginning of the next trial.

Individual fits

We have focused on group-level fits because we are especially interested in the ability of the model to predict differences between binary and trinary decisions. However, it is important to verify that the qualitative effects that we emphasize also hold in individual data, and are not aggregation artifacts. It is also interesting to see to what extent the model can account for individual variability in fixation and choice behavior. To address both of these concerns, we present versions of each plot shown in the main text with separate panels for each participant. The model was fit to each participant's data following the same fitting procedure as for the group-level fit (using the same precomputed likelihood histograms). Finally, because many of the behavioral patterns are quite noisy with only 50 trials, we aditionally plot Bayesian linear model fits for both the human and model-simulated data (using logistic regression for binary dependent variables). These predictions were generated using the rstanarm package [3]. The plots are included in the S2 Appendix.

In brief, we found that most behavioral patterns shown in the main text figures were consistently demonstrated by a majority of participants. However, although most effects were consistently present and in the correct direction, the strength often varied considerably across individuals. In many cases, the model showed only a modest ability to capture this variability. This reflects the strong *a priori* assumptions of the model, in particular, the assumption that attention is allocated optimally.

Parameter recovery

To validate our model fitting approach, we conducted a parameter recovery exercise. We began by sampling 1024 "true" parameter configurations from the promising region of the parameter space that we considered when fitting human data (see main text *Methods*). We sampled these values using the 5-dimensional Sobol sequence [4] to ensure good coverage of the space. For each parameter configuration, we computed two sets of 80 near-optimal policies (one for binary choice and one for trinary choice) using the UCB-based method described in the main text. Then, for each set, we simulated the even trials of the corresponding dataset. We simulated each trial only once (to match the amount of data when fitting participants), cycling between the 80 near-optimal policies. We then applied the full approximate maximum likelihood estimation procedure described in the main text for each dataset.[1] For each configuration, the maximum likelihood estimate of each parameter was its mean in the 30 configurations with highest likelihood (following our reporting approach for the fits to human data).

The results, shown in Figure A, suggest that we were able to recover parameters with fairly high accuracy. For all parameters besides the softmax temperature, the Pearson correlation was over 0.9. Importantly, we found only slight bias in the estimation procedure, with the best fitting linear regression line falling close to the equality line for all parameters. The largest bias was for the prior bias parameter, α, for which the recovered parameter was on average .095 less than the true parameter.

To validate our approach when fitting individual subjects, we repeated the steps above, except using only 50 simulated trials (the number of fitting trials for each subject). Unsurprisingly, we find that the estimates become less reliable; however the correlations are still fairly strong. In the trinary case, we see substantial bias for both γ_{sample} and α. Thus, care must be taken when interpreting the individual fitting results.

Implementation and validation of the aDDM

In order to compare our model to the predictions of the aDDM [1,2], we reimplemented it based on code provided from the first author. We made one change to the simulation

[1] We reused the likelihood histograms that we computed when fitting participant data. Critically, however, the policies used to generate these histograms were not the same ones used to generate the simulated data.

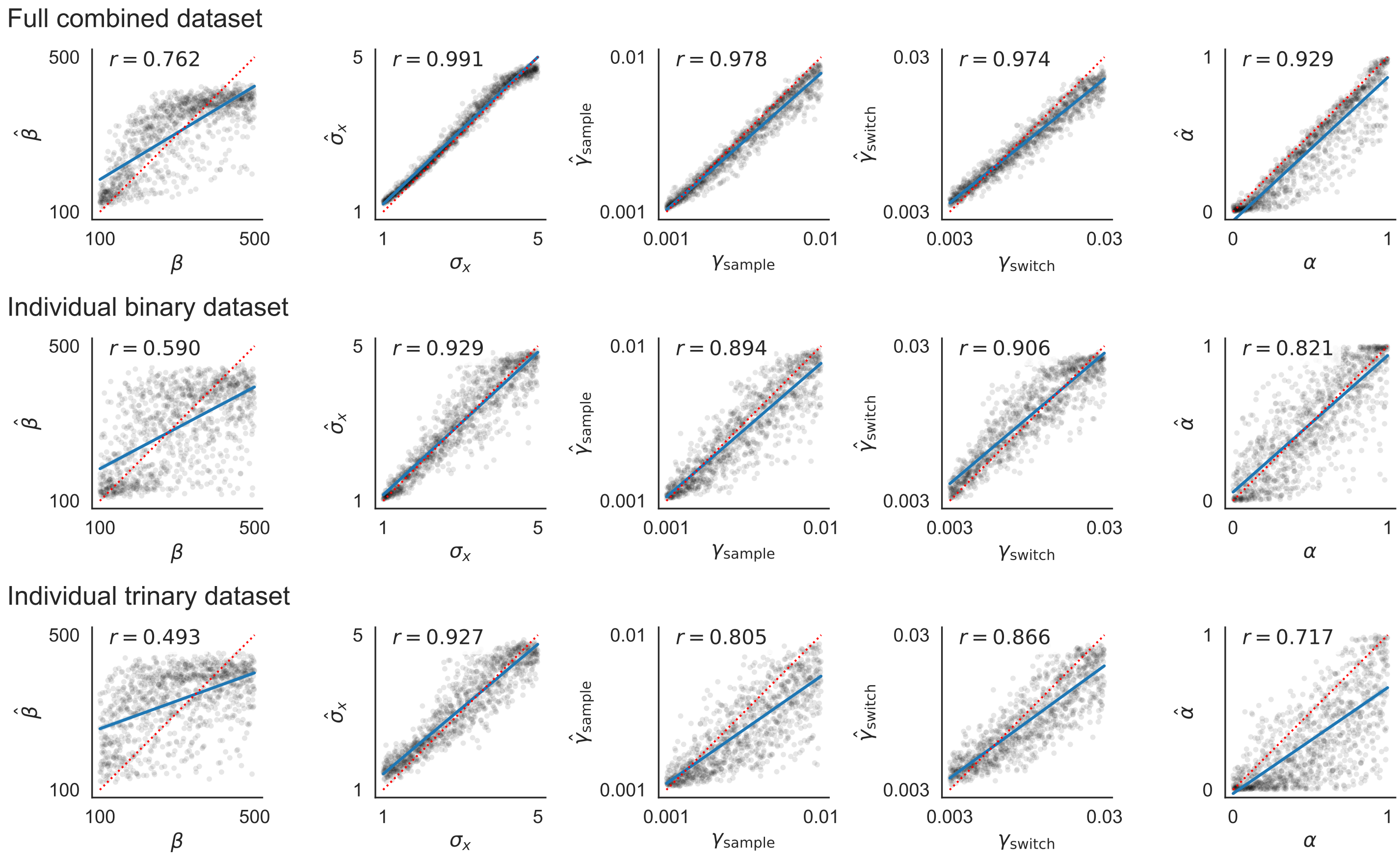

Figure A: Parameter recovery. Each panel plots the estimated parameter value as a function of the true parameter value. Each black dot corresponds to one simulated dataset. The dotted red line shows equality (i.e., perfect recovery) and the solid blue line shows the linear trend. The top row shows results when simulating the full joint dataset. The middle row shows results when simulating 50 trials (the amount of fitting data one individual produces) of binary choice. The bottom row shows the same for trinary choice.

procedure. In the original papers, the model predictions were generated by simulating an equal number of trials for all possible combinations of item ratings. In contrast, we have simulated each trial in the dataset a fixed number of times. That is, our simulations follow the empirical distribution of the item ratings. To verify the correctness of our implementation, we have replicated four key plots from the original binary and trinary papers, shown in Figures B and B respectively. Note that for these plots, we use the original approach of simulating each possible combination a fixed number of times.

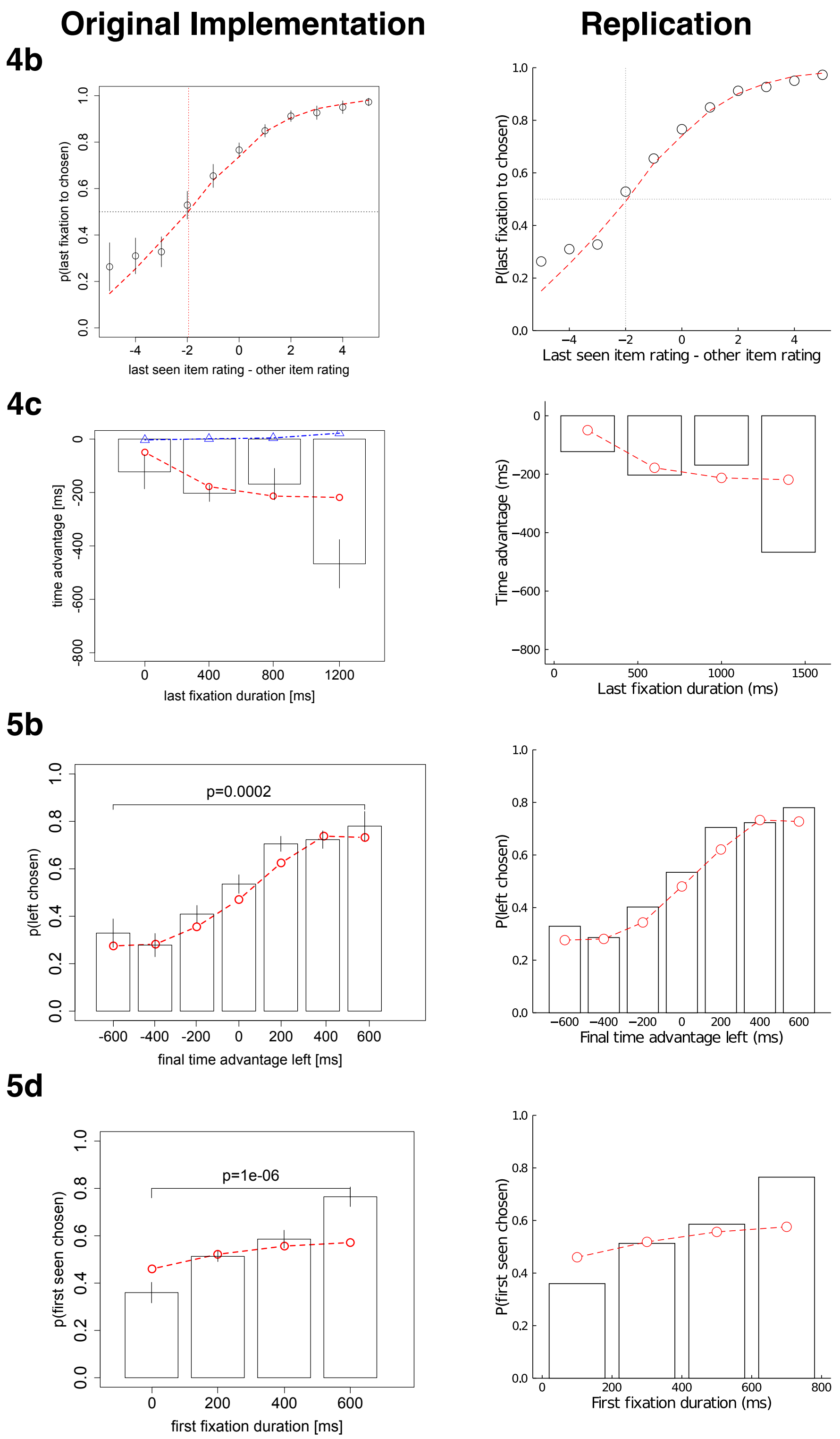

Figure B: Replication of Krajbich, Armel, and Rangel (2010). Note that x axis labels in the orginal plots sometimes reflected the left tail of the bin; in these cases, we adjusted the tick locations accordingly.

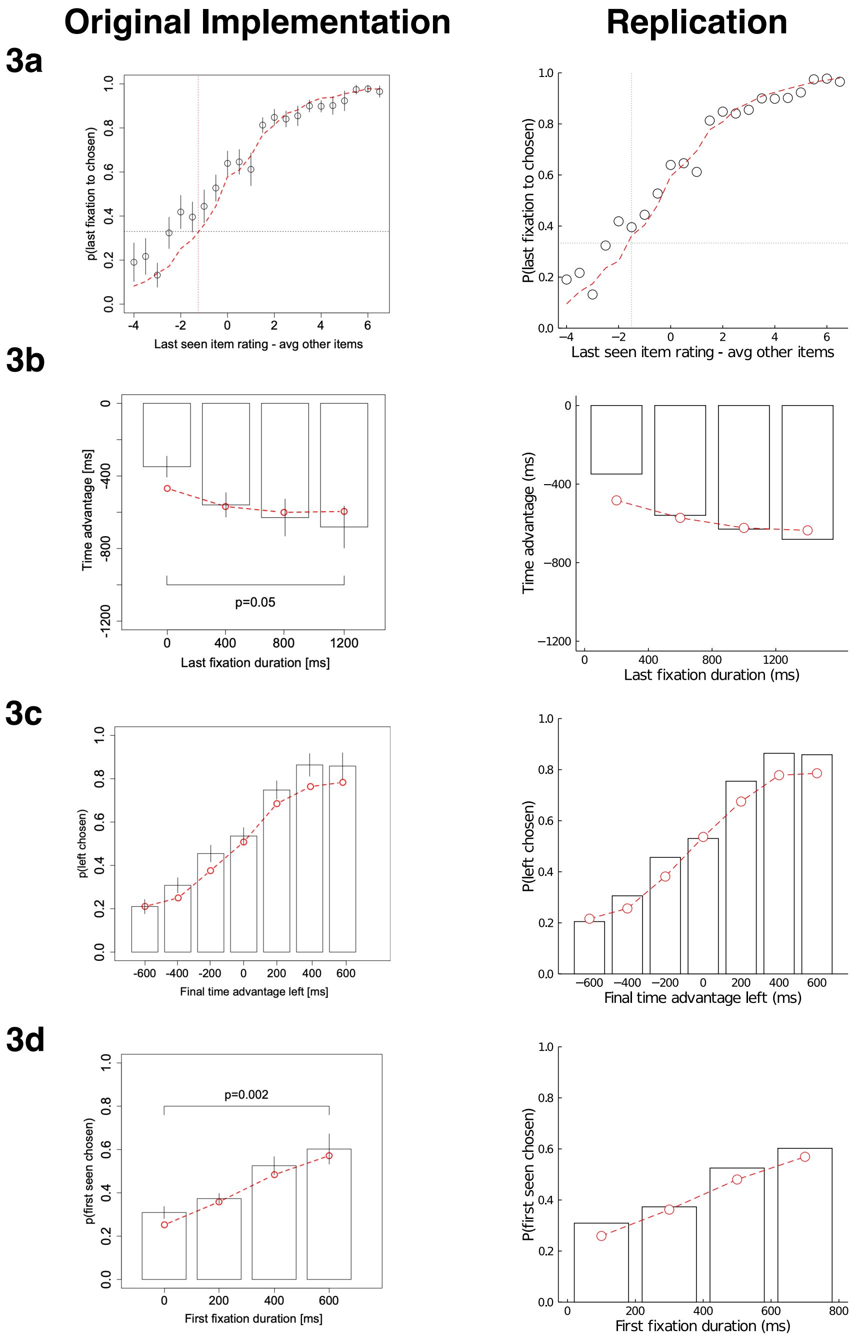

Figure C: Replication of Krajbich and Rangel (2011). Note that there are slight deviations in model predictions due to noise in the simulations; the orginal plots are based on 2000 simulated trials.

Derivation: Myopic Value of Information

The myopic value of information is the value of the information acquired by a single computation, that is, the expected increase in decision quality from executing a single computation and then deciding rather than making a decision immediately. Formally,

$$\mathrm{VOI}_{\mathrm{myopic}}(b_t, c) = \mathrm{E}_{b_{t+1}|b_t,c}\left[R(b_{t+1}, \perp)\right] - R(b, \perp).$$

In our model, this is equal to the expected value of the item that will be chosen after taking an additional sample minus the expected value of an item chosen based on the current beliefs. That is,

$$\mathrm{VOI}_{\mathrm{myopic}}(b_t, c) = \mathrm{E}_{\mu_{t+1}|\mu_t,\lambda_t}\left[\max_i \mu_{t+1}^{(i)}\right] - \max_i \mu_t^{(i)}.$$

Because μ_{t+1} differs from μ_t only for item c, we can rewrite the expectation term as

$$\mathrm{E}_{\mu_{t+1}^{(c)}|\mu_t^{(c)},\lambda_t^{(c)}}\left[\max\left\{\mu_{t+1}^{(c)}, \max_{i\neq c}\mu_t^{(i)}\right\}\right]. \tag{1}$$

Thus, the term inside the expectation is the maximum of a constant, $\max_{i\neq c}\mu_t^{(i)}$, and a univariate random variable, $\mu_{t+1}^{(c)} \mid \mu_t^{(c)}, \lambda_t^{(c)}$. To simplify notation, we suppress the conditioning variables in the following derivation.

To derive an analytic expression for Equation [1], we first derive the distribution of $\mu_{t+1}^{(c)}$, that is, the distribution over the posterior mean after taking a sample. Applying the transition dynamics given in Equation **??**, we have

$$\mu_{t+1}^{(c)} = \frac{\sigma_x^{-2}x_t + \lambda_t^{(c)}\mu_t^{(c)}}{\lambda_t^{(c)} + \sigma_x^{-2}}. \tag{2}$$

Since $x_t \mid u^{(c)} \sim \mathrm{Gaussian}(u^{(c)}, \sigma_x^2)$ and $\mu_{t+1}^{(c)}$ is a linear transformation of x_t, it follows that $\mu_{t+1}^{(c)}$ is a Gaussian random variable. Additionally, because the belief is a distribution

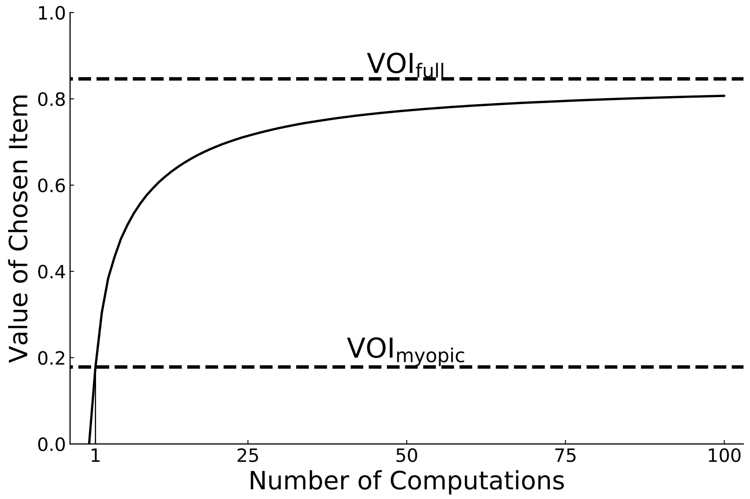

Figure D: Illustration of the value of information features. The solid line shows the average value of the item chosen after different numbers of computations selected by a near-optimal policy assuming no computational costs. The dashed lines show values for two of the VOI features in the initial belief state: VOI$_{\mathrm{myopic}}$ is the value after one computation and VOI$_{\mathrm{full}}$ is the asymptotitc value after infinite computations.

over the true utility, we have $u^{(c)} \mid \mu_t^{(c)}, \lambda_t^{(c)} \sim \text{Gaussian}(\mu_t^{(c)}, 1/\lambda_t^{(c)})$. Combining these two statements, we see that $x_t \mid \mu_t^{(c)}, \lambda_t^{(c)}$ is a Gaussian whose mean is itself a Gaussian. Applying the fact that $\text{Gaussian}(\mu, \sigma^2) = \mu + \text{Gaussian}(0, \sigma^2)$, we can then derive that $x_t \mid \mu_t^{(c)}, \lambda_t^{(c)} \sim \text{Gaussian}(\mu_t^{(c)}, {}^1\!/\lambda_t^{(c)}) + \text{Gaussian}(0, \sigma_x)$, which reduces to $\text{Gaussian}(\mu_t^{(c)}, {}^1\!/\lambda_t^{(c)} + \sigma_x^2)$. Finally, applying the linear transformation of x_t given by Equation 2, we have

$$\mu_{t+1}^{(c)} \sim \text{Gaussian}(\mu_\mu, \sigma_\mu^2)$$

where

$$\mu_\mu = \frac{\sigma_x^{-2}}{\lambda_{t+1}^{(c)}} \mu_t^{(c)} + \frac{\lambda_t^{(c)} \mu_t^{(c)}}{\lambda_{t+1}^{(c)}} = \mu_t^{(c)}$$

and

$$\sigma_\mu^2 = \left(\frac{\sigma_x^{-2}}{\lambda_{t+1}^{(c)}} \right)^2 \left(\frac{1}{\lambda_t^{(c)}} + \sigma_x^2 \right).$$

Having derived the distribution of Equation $\mu_{t+1}^{(c)}$, we now turn to the expected maximum in [1]. From basic probability theory we know that for any constant z and random variable X,

$$\text{E}[\max\{X, z\}] = \Pr[X \leq z] \cdot z + (1 - \Pr[X \leq z]) \cdot \text{E}[X \mid X > z]. \tag{3}$$

Substituting $\max_{i \neq c} \mu_t^{(i)}$ for z and $\mu_{t+1}^{(c)}$ for X, we can use this formula to derive an analytical solution for the myopic value of information. First, we have

$$\Pr\left[\mu_{t+1}^{(c)} \leq \max_{i \neq c} \mu_t^{(i)} \right] = \Phi(\beta),$$

where Φ is the cumulative density function (CDF) of a standard Gaussian , and

$$\beta = \frac{\max_{i \neq c} \mu_t^{(i)} - \mu_\mu.}{\sigma_\mu}$$

Next, we apply the standard formula for the expectation of a truncated Gaussian, giving us

$$E\left[\mu_{t+1}^{(c)} \mid \mu_{t+1}^{(c)} > \max_{i \neq c} \mu_t^{(i)} \right] = \mu_\mu + \frac{\phi(\beta)}{1 - \Phi(\beta)} \sigma_\mu,$$

where ϕ is the standard normal probability density function. Finally, putting this together we find that $\text{VOI}_{\text{myopic}}(b, c)$ is equal to

$$\Phi(\beta) \cdot \max_{i \neq c} \mu_t^{(i)} + (1 - \Phi(\beta)) \cdot \left(\mu_\mu + \frac{\phi(\beta)}{1 - \Phi(\beta)} \sigma_\mu \right) - \max_i \mu_t^{(i)}.$$

Derivation: Value of Perfect Information About One Item

Whereas $\text{VOI}_{\text{myopic}}$ captures the information value of a single sample, VOI_{item} captures the information value of an infinite number of samples for one item, that is, the value of knowing the exact value of one item. Formally,

$$\text{VOI}_{\text{item}}(b_t, c) = \text{E}_{u^{(c)} \mid \mu_t^{(c)}, \lambda_t^{(c)}} \left[\max\left\{ u^{(c)}, \max_{i \neq c} \mu_t^{(i)} \right\} \right] - \max_i \mu_t^{(i)}.$$

The derivation is similar to that of $\text{VOI}_{\text{myopic}}$, but instead of taking the expectation over the posterior mean after one computation, $\mu_{t+1}^{(c)}$, we take the expectation over the true utility,

$u^{(c)} \mid \mu_t^{(c)}, \lambda_t^{(c)} \sim \text{Gaussian}(\mu_t^{(c)}, 1/\lambda_t^{(c)})$. Thus, we apply the same steps beginning with [3], but replacing $\mu_{t+1}^{(c)}$ with $u^{(c)} \mid \mu_t^{(c)}, \lambda_t^{(c)}$. This results in $\text{VOI}_{\text{item}}(b, c)$ equal to

$$\Phi(\beta') \cdot \max_{i \neq c} \mu_t^{(i)} + (1 - \Phi(\beta')) \cdot \left(\mu_t^{(c)} + \frac{\phi(\beta')}{1 - \Phi(\beta')} \sqrt{1/\lambda_t^{(c)}} \right) - \max_i \mu_t^{(i)}$$

where

$$\beta' = \frac{\max_{i \neq c} \mu_t^{(i)} - \mu_t^{(c)}}{\sqrt{1/\lambda_t^{(c)}}}.$$

Derivation: Value of Perfect Information About All Items

VOI_{full} captures the information value of learning the exact value of every item in the choice set, that is acquiring full information. In this case, the DM will make an exactly optimal choice, gaining the utility of the item that is in fact best. Formally,

$$\text{VOI}_{\text{full}}(b) = \mathrm{E}_{u \mid \mu_t^{(c)}, \lambda_t^{(c)}} \left[\max_i \left\{ u^{(i)} \right\} \right] - \max_i \mu_t^{(i)}. \tag{4}$$

For the case of N items, the conditional expectation term is given by the integral

$$\int \ldots \int \left[\max_i u^{(i)} \prod_{i=1}^{k} \text{Gaussian}(u^{(i)}; \mu_t^{(i)}, 1/\lambda_t^{(i)}) \right] du^{(1)} \ldots du^{(N)}.$$

Unfortunately, there is no analytic solution to this integral. However, we can substantially reduce our computational burden by reducing to a piecewise one-dimensional integral. First, we can express the expectation of any random variable as a piecewise integral,

$$\mathrm{E}[X] = -\int_{-\infty}^{0} F_X(x)\, dx + \int_{0}^{\infty} (1 - F_X(x))\, dx, \tag{5}$$

where F_X is the CDF of X. Next, we can express the CDF of the maximum of a set of random variables as the product of the CDF for each variable alone,

$$F_{\max \mathcal{X}}(x) = \prod_{X \in \mathcal{X}} F_X(x), \tag{6}$$

because the maximum of a set is less than x if and only if each element in the set is less than x. In our case, the set χ contains the belief distributions for each item. Letting M denote $\max \mathcal{X}$, we can define its CDF as

$$F_M(m) = \prod_{i=1}^{N} \Phi\left(\sqrt{\lambda_t^{(i)}} \left(m - \mu_t^{(i)} \right) \right).$$

Combining Equations [4], [5], and [6], we arrive at the following expression for $\text{VOI}_{\text{full}}(b)$:

$$-\int_{-\infty}^{0} F_M(x)\, dx + \int_{0}^{\infty} (1 - F_M(x))\, dx - \max_i \mu_t^{(i)}.$$

We evaluate these two integrals numerically to a minimum precision of 10^{-5} by the adaptive Gauss-Kronrod quadrature method implemented in the QuadGK Julia package.

Despite the dimensionality reduction, we found that evaluating these integrals was still the primary computational bottleneck for simulating the model. Thus, in order to reduce computation time, we only compute VOI_{full} when it is necessary to determine which computation the policy will execute. As detailed below, this is often unnecessary because the other features already determine which feature has maximal $\widehat{\text{VOC}}$.

Critically, the modification that we describe here has no effect on the behavior of the policy or the predictions of the models; we have verified this assertion through simulation.

This computational trick is based on three insights. First, note that VOI_{full} helps to decide whether or not to take another sample, but not which item to sample from. Thus, we can determine which computation the policy would take, conditional on taking a sample at all, based only on the $\text{VOI}_{\text{myopic}}$ and VOI_{item} features. Given that these two features have an analytical solution, as derived above, we can quickly identity the best item to sample from, which is given by

$$c^* = \arg\max_{c \neq \perp} \left\{ w_1 \cdot \text{VOI}_{\text{myopic}}(b, c) + w_2 \cdot \text{VOI}_{\text{item}}(b, c) - \text{cost}(c) + w_4 \right\}. \tag{7}$$

Second, since $\text{VOC}(b, \perp) = 0$, it follows that if $\widehat{\text{VOC}}(b, c^*) > 0$, the policy should sample from item c^*, and otherwise it should stop sampling. In general, determining the sign of $\widehat{\text{VOC}}(b, c^*)$requires evaluating $\text{VOI}_{\text{full}}(b)$. However, in some cases the sign can be determined without knowing $\text{VOI}_{\text{full}}(b)$. In particular, we can take advantage of the fact that $\text{VOI}_{\text{item}}(b, c) \leq \text{VOI}_{\text{full}}(b)$ for all b, c. We can thus compute a lower bound on $\widehat{\text{VOC}}(b, c)$ by replacing $\text{VOI}_{\text{full}}(b)$ with $\text{VOI}_{\text{item}}(b, c)$ in Equation [7]. If this lower bound is positive, then we know the full approximation would also be positive, and thus the optimal choice is to sample from item c^*. Otherwise, we compute $\text{VOI}_{\text{full}}(b)$ and identify the optimal computation using all of the features.

Third, at first sight this approach might seem to be incompatible with the soft-maximizing policy, where computation c is selected with probability proportional to $\exp \beta \widehat{\text{VOC}}(b, c)$. In particular, the standard method for sampling from this distribution requires fully evaluating $\widehat{\text{VOC}}(b, c)$. However, we can circumvent this issue using the Gumbel-max trick [5], which provides a way to sample from a Boltzman (softmax) distribution by taking the argmax of the unexponentiated values corrupted by Gumbel noise. Formally,

$$\Pr\left[\arg\max_i \{x_i + \epsilon_i\} = j\right] = \frac{\exp x_j}{\sum_i \exp x_i}.$$

As a result, we can rewrite the soft-max policy as

$$\pi(b; \mathbf{w}, \beta) = \arg\max_c \left\{ \beta \widehat{VOC}(b, c; w) + \epsilon_c \right\},$$

where $\epsilon_c \sim \text{Gumbel}(0, 1)$. We can then implement steps 1 and 2 of the short-cut, adding ϵ_c to the right hand side of Equation 7, and comparing the lower-bound VOC to an independent Gumbel sample, $\epsilon_\perp$, rather than 0 to capture the noise applied to $\text{VOC}(b, \perp)$.

Quality of the approximation method in Bernoulli model

The approximation method used here has previously been shown to learn policies with near-optimal performance on a metalevel MDP similar to the one in the present model, but with Bernoulli-distributed samples and *no* switching costs [6]. The logic of the problem is identical: A DM wants to select the best item and informs her decision by drawing noisy samples with an expected value equal to the items' true utility. However, in the simpler Bernoulli case that has been previously studied, true utilities take values between 0 and 1, samples from item c are drawn from $\text{Bernoulli}(u^{(c)})$, and the uniform distribution over all possible utilities, $\text{Beta}(1, 1)$, provides a conjugate prior. Thus, posterior beliefs take the form $\text{Beta}(1 + a, 1 + b)$, where a and b are respectively the number of times 1 and 0 have been sampled for the given item. Critically, the resulting belief space is discrete because a and b are integers. This allows the computation of the exact optimal policy by dynamic programming, if an upper bound on the number of samples that can be taken is assumed.

Callaway et al. [6] take advantage of this fact to show that the policy approximation method used here provides a highly-accurate approximation of the optimal policy. However, their model does not have switching costs, which could potentially make the approximation perform much worse. Here, we investigate this issue by adding switching costs to

the Bernoulli model, and measuring their impact on the method's performance. Ideally we would be able to directly assess the performance of our method in the full model with Gaussian samples, but an optimal solution for this case is not available and deriving one is beyond the scope of the study.

To aid interpretation, we re-parameterized the switching cost as $\gamma_{\text{switch}} = (k-1)\gamma_{\text{sample}}$ such that k can be interpreted as a multiplier on the base sample cost. For example, $k = 1$, indicated by "1x" in the figure, corresponds to no switching cost. We considered a grid of cost parameters with $\gamma_{\text{sample}} \in \{e^{-9}, e^{-8}, \ldots, e^{-3}\}$ and $k \in 1, 2 \ldots, 10$. We set an upper bound of 75 samples. As shown in Fig. E, we replicated previous results that the approximated policy is nearly optimal when there is no switch cost. As the figure shows, relative performance degrades somewhat when switch costs are added, but the approximation still achieves 92% of the optimal metalevel reward in the worst case explored.

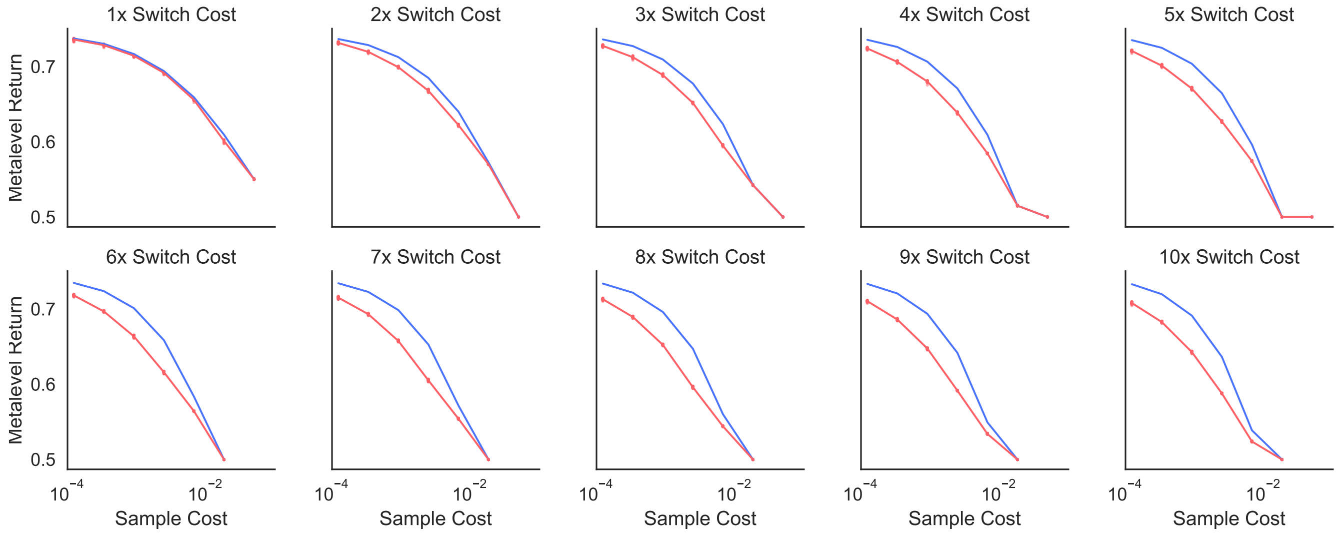

Figure E: Performance of the policy approximation (red) compared with the true optimal solution (blue) on the Bernoulli model with switching costs. The red line shows mean performance from the top 80 policies identified by the UCB algorithm. Additionally each individual policy's performance is plotted as an individual point, but performance is so consistent that the points are not visually distinct.

References

1. Krajbich I, Armel C, Rangel A. Visual fixations and the computation and comparison of value in simple choice. Nature Neuroscience. 2010;13(10):1292–1298.

2. Krajbich I, Rangel A. Multialternative drift-diffusion model predicts the relationship between visual fixations and choice in value-based decisions. Proceedings of the National Academy of Sciences. 2011;108(33):13852–13857.

3. Goodrich B, Gabry J, Ali I, Brilleman S. rstanarm: Bayesian applied regression modeling via Stan.; 2020. Available from: `https://mc-stan.org/rstanarm`.

4. Sobol' IM. On the distribution of points in a cube and the approximate evaluation of integrals. Zhurnal Vychislitel'noi Matematiki i Matematicheskoi Fiziki. 1967;7(4):784–802.

5. Yellott Jr JI. The relationship between Luce's choice axiom, Thurstone's theory of comparative judgment, and the double exponential distribution. Journal of Mathematical Psychology. 1977;15(2):109–144.

6. Callaway F, Gul S, Krueger P, Griffiths TL, Lieder F. Learning to select computations. In: Uncertainty in Artificial Intelligence: Proceedings of the Thirty-Fourth Conference; 2018.
