# Supplementary material for: Fixation patterns in simple choice reflect optimal information sampling
Source: PLoS Comput Biol. 2021 Mar 26;17(3):e1008863. doi: 10.1371/journal.pcbi.1008863 (PMC8026028; doi:10.1371/journal.pcbi.1008863)
Supplement: S2 Appendix — Includes versions of all plots in the main text with separate panels for each participant (including model predictions with parameters fit to each participant). (PDF) [file pcbi.1008863.s002.pdf]

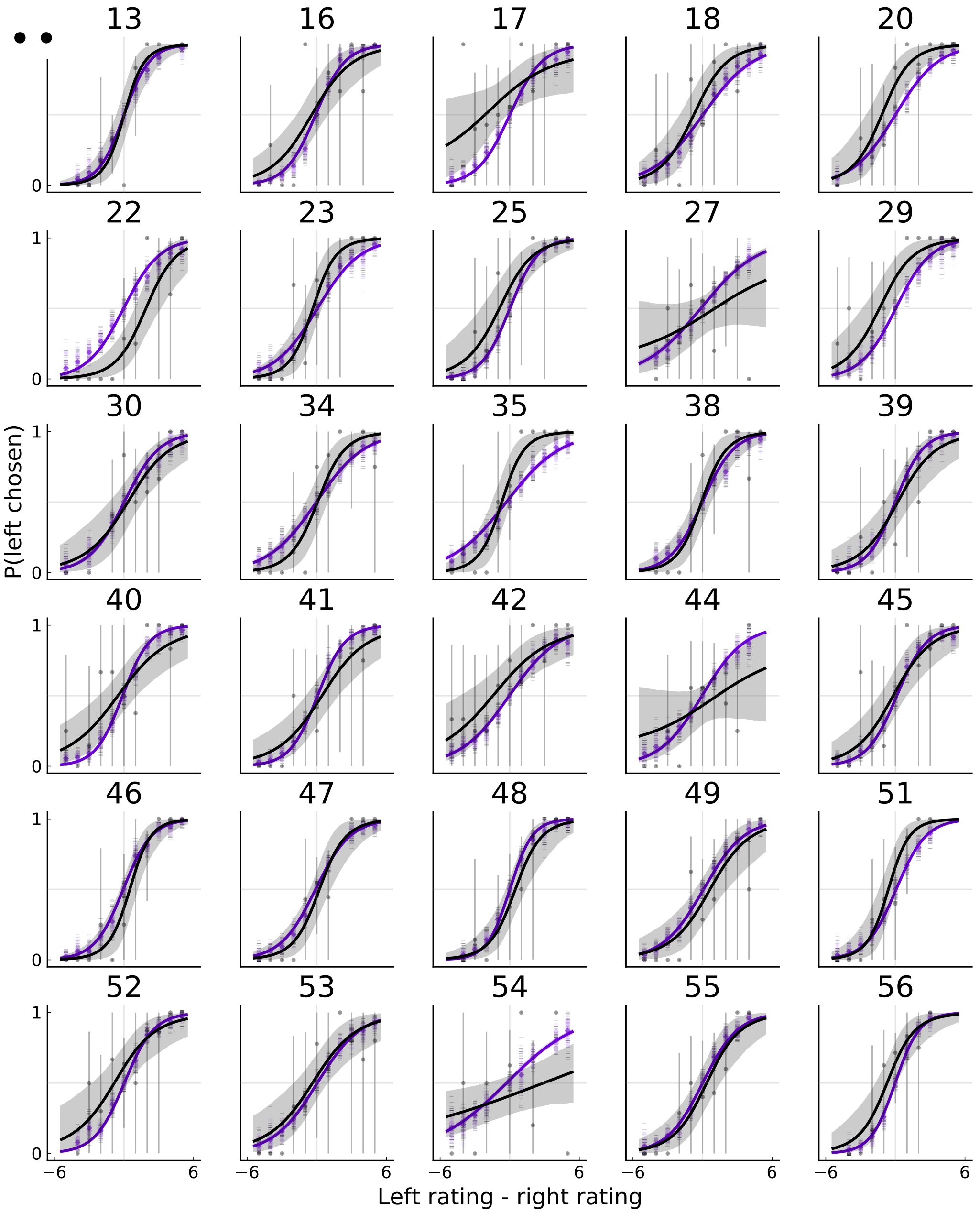

Figure A: Individual fits for binary dataset, Figure 3A: Choice probability as a function of relative rating. Each panel shows data and model predictions for one participant. Raw human and model-simulated data are shown as gray dots and purple diamonds. For human data, error bars show 95% confidence intervals. For the model, each of the top 30 parameter configurations are shown in light purple and the mean is shown in dark purple (confidence intervals excluded for legibility). The solid lines shows the posterior predictive mean of a Bayesian linear model; the shaded area denotes the 95% credible interval (often invisible for the model).

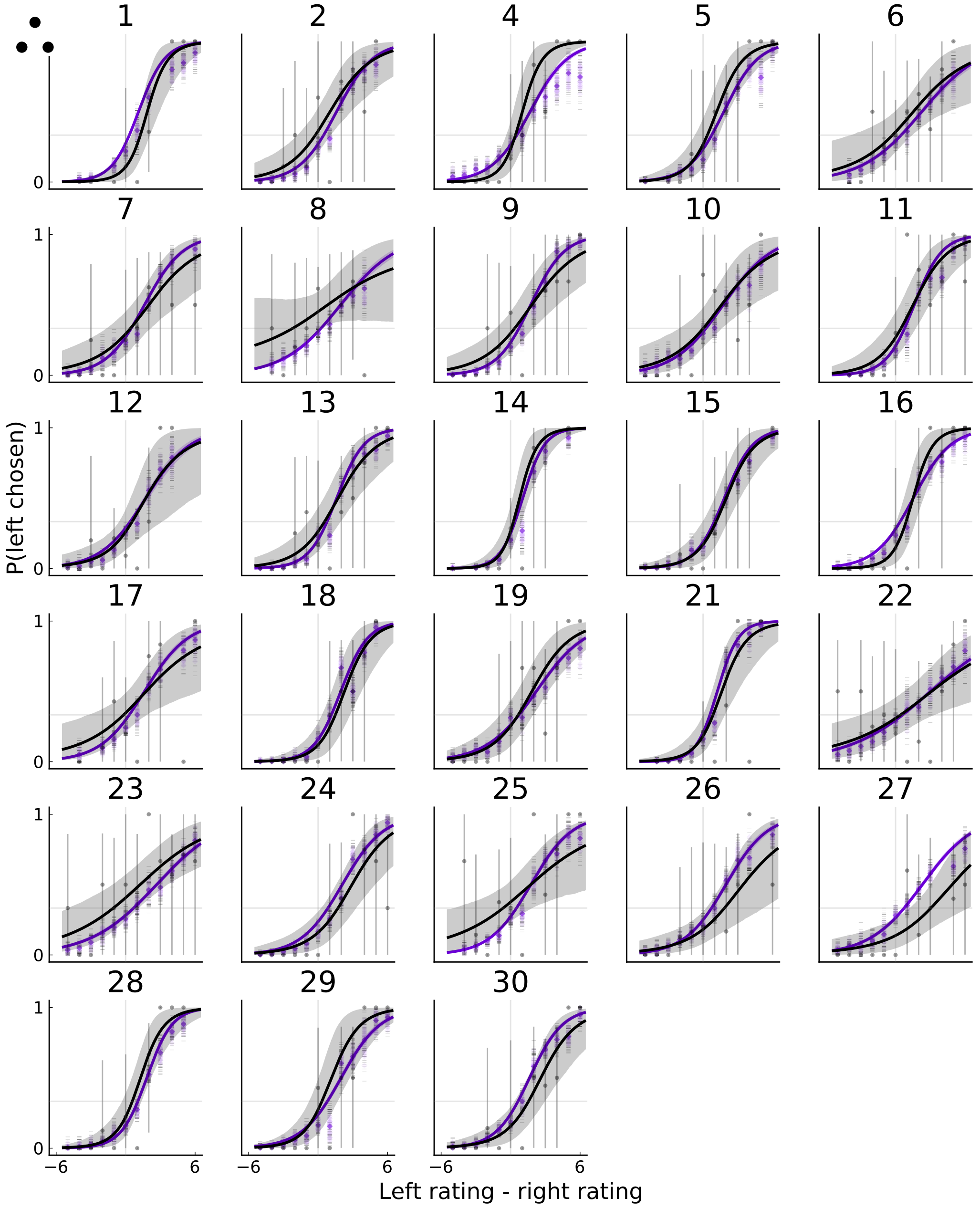

Figure B: Individual fits for trinary dataset, Figure 3A: Choice probability as a function of relative rating. Curves show the predictive mean and 95% confidence intervals of a Bayesian linear model. Human data are in black and model predictions are in purple. See the caption of Figure A for details.

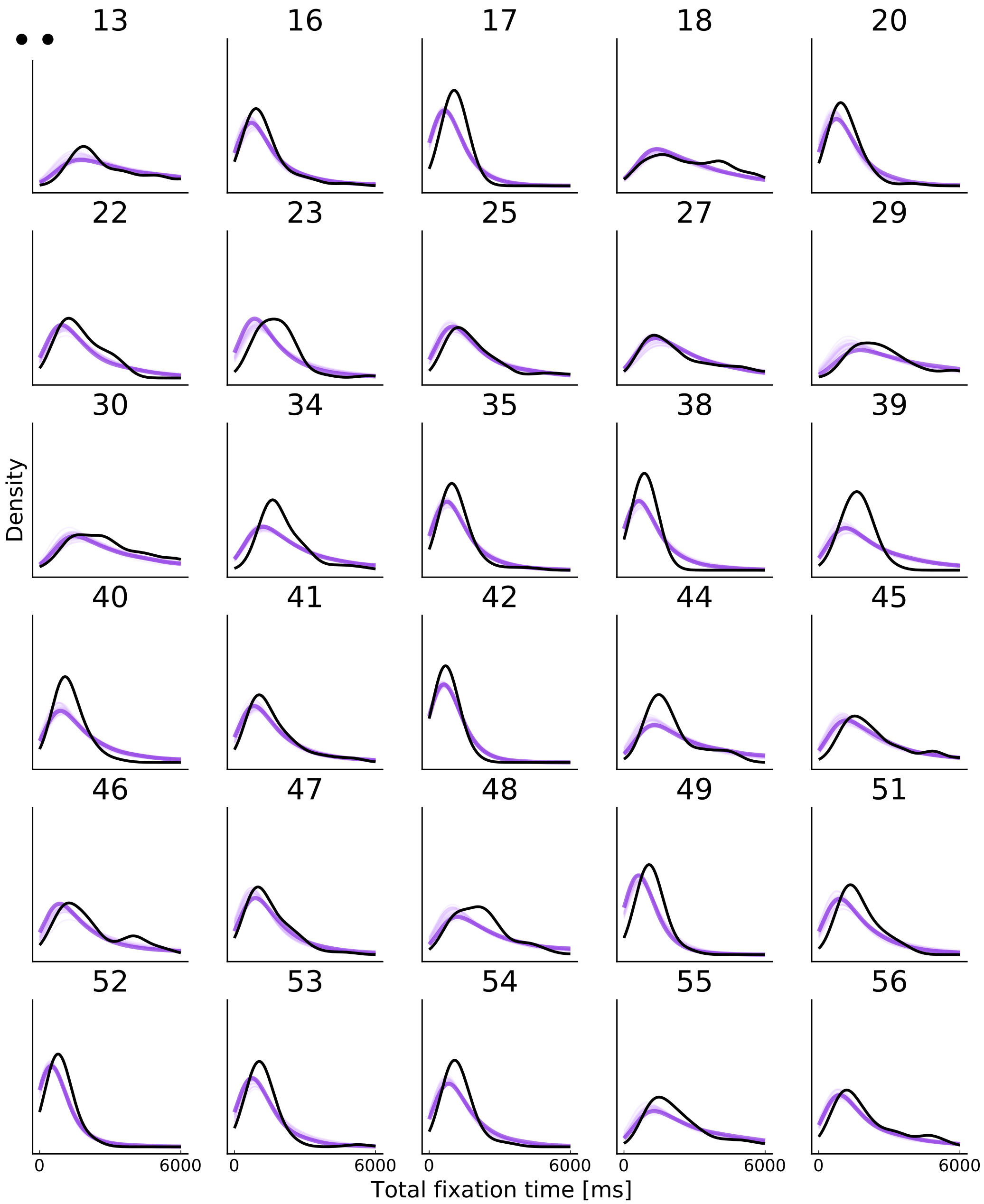

Figure C: Individual fits for binary dataset, Figure 3B: Kernel density estimation for the distribution of total fixation time. Light purple shows predictions of the top 30 parameter configurations. Dark purple shows the aggregate prediction. Human data are in black and model predictions are in purple. See the caption of Figure A for details.

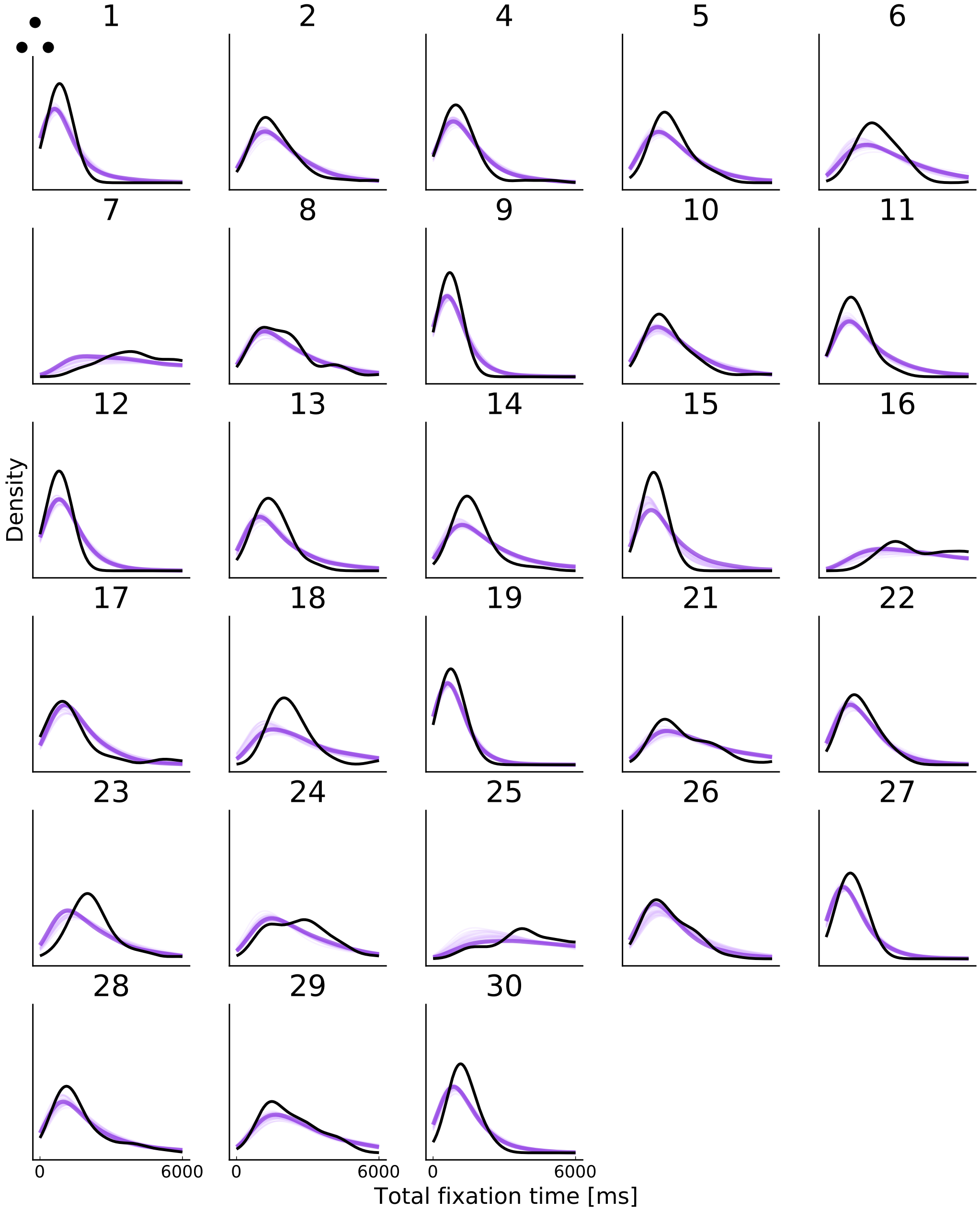

Figure D: Individual fits for trinary dataset, Figure 3B: Kernel density estimation for the distribution of total fixation time. Light purple shows predictions of the top 30 parameter configurations. Dark purple shows the aggregate prediction. Human data are in black and model predictions are in purple. See the caption of Figure A for details.

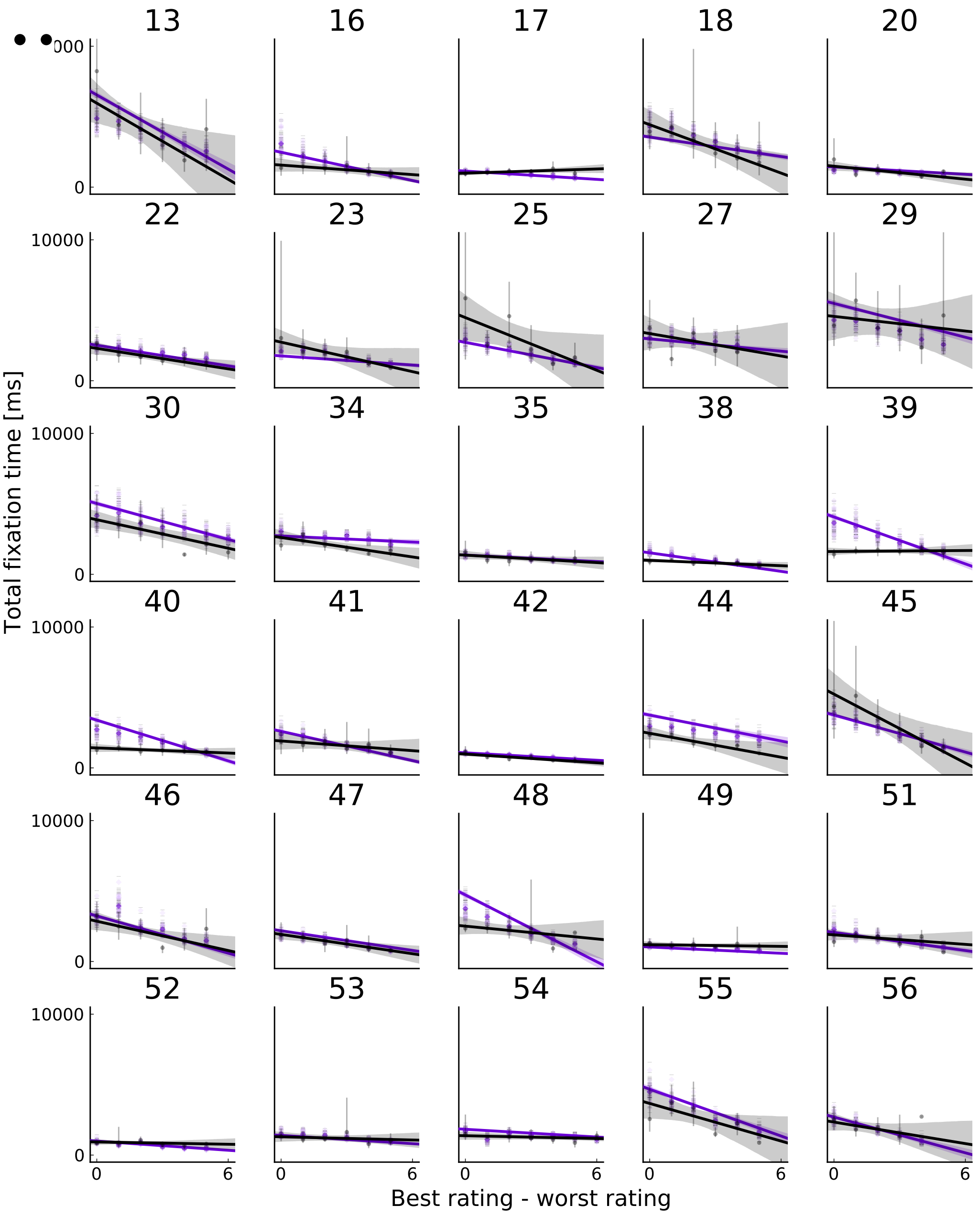

Figure E: Individual fits for binary dataset, Figure 3C: Total fixation time as a function of the relative rating of the highest rated item. Curves show the predictive mean and 95% confidence intervals of a Bayesian linear model. Human data are in black and model predictions are in purple. See the caption of Figure A for details.

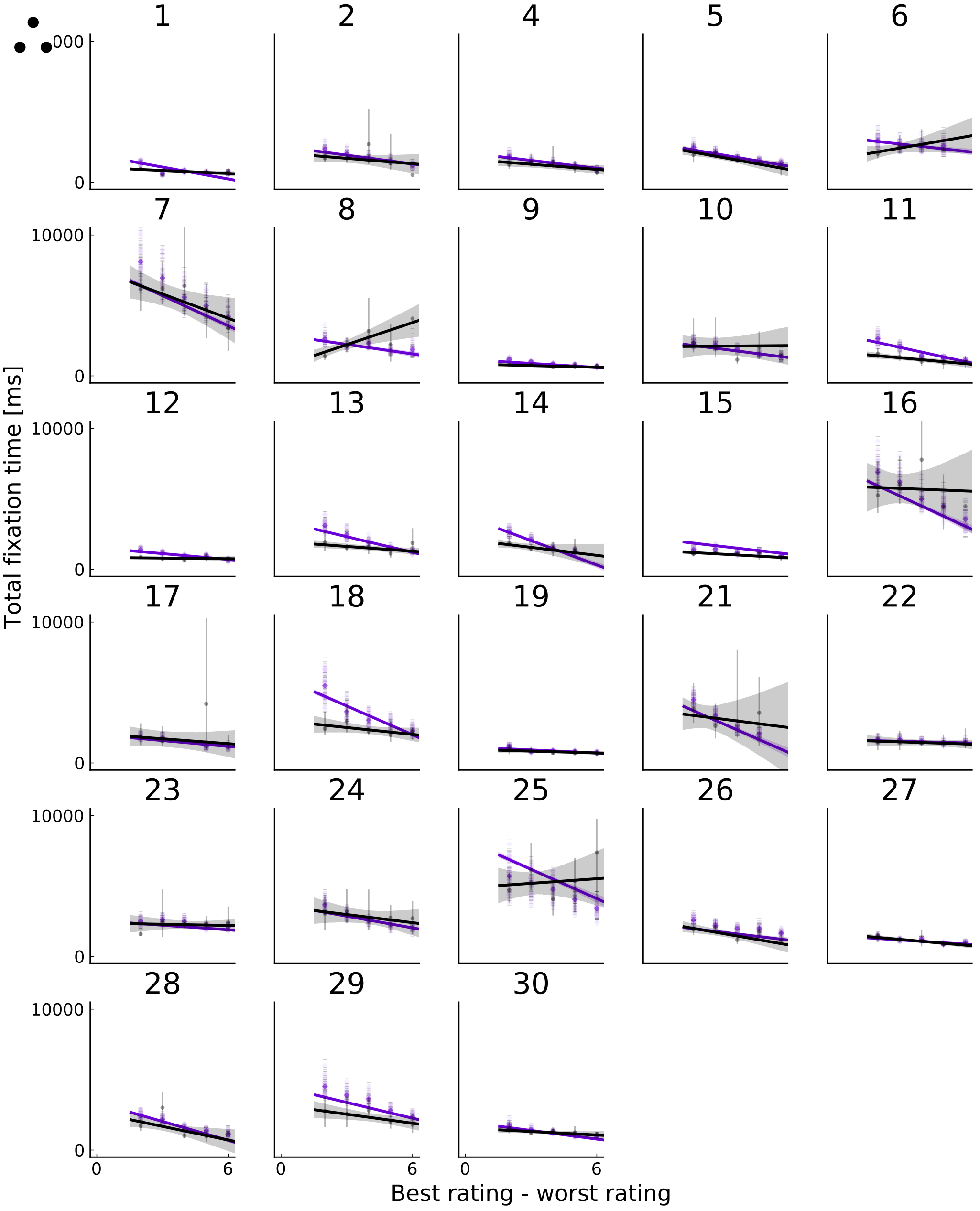

Figure F: Individual fits for trinary dataset, Figure 3C: Total fixation time as a function of the relative rating of the highest rated item. Curves show the predictive mean and 95% confidence intervals of a Bayesian linear model. Human data are in black and model predictions are in purple. See the caption of Figure A for details.

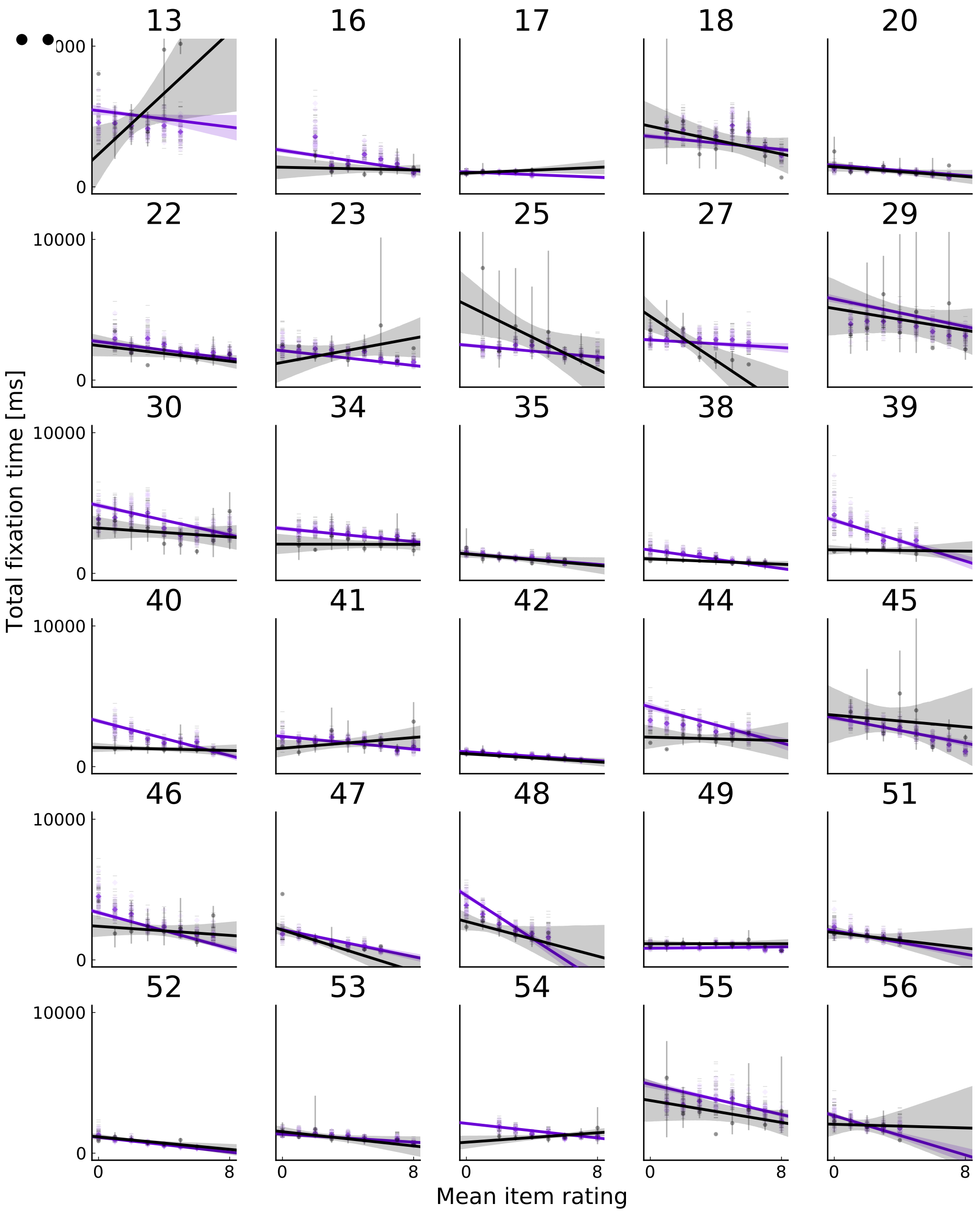

Figure G: Individual fits for binary dataset, Figure 3D: Total fixation time as a function of the mean of all the item ratings (overall value). Curves show the predictive mean and 95% confidence intervals of a Bayesian linear model. Human data are in black and model predictions are in purple. See the caption of Figure A for details.

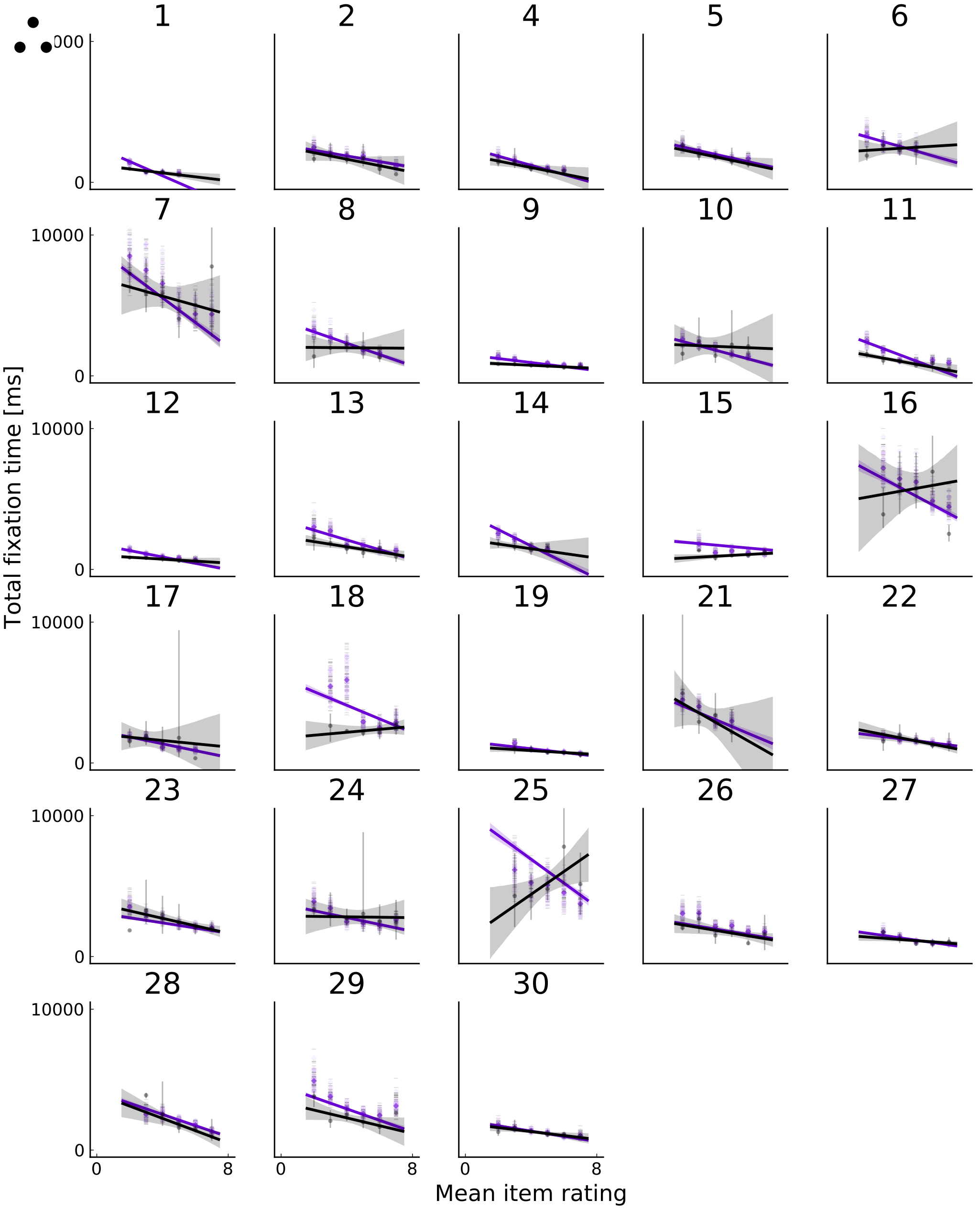

Figure H: Individual fits for trinary dataset, Figure 3D: Total fixation time as a function of the mean of all the item ratings (overall value). Curves show the predictive mean and 95% confidence intervals of a Bayesian linear model. Human data are in black and model predictions are in purple. See the caption of Figure A for details.

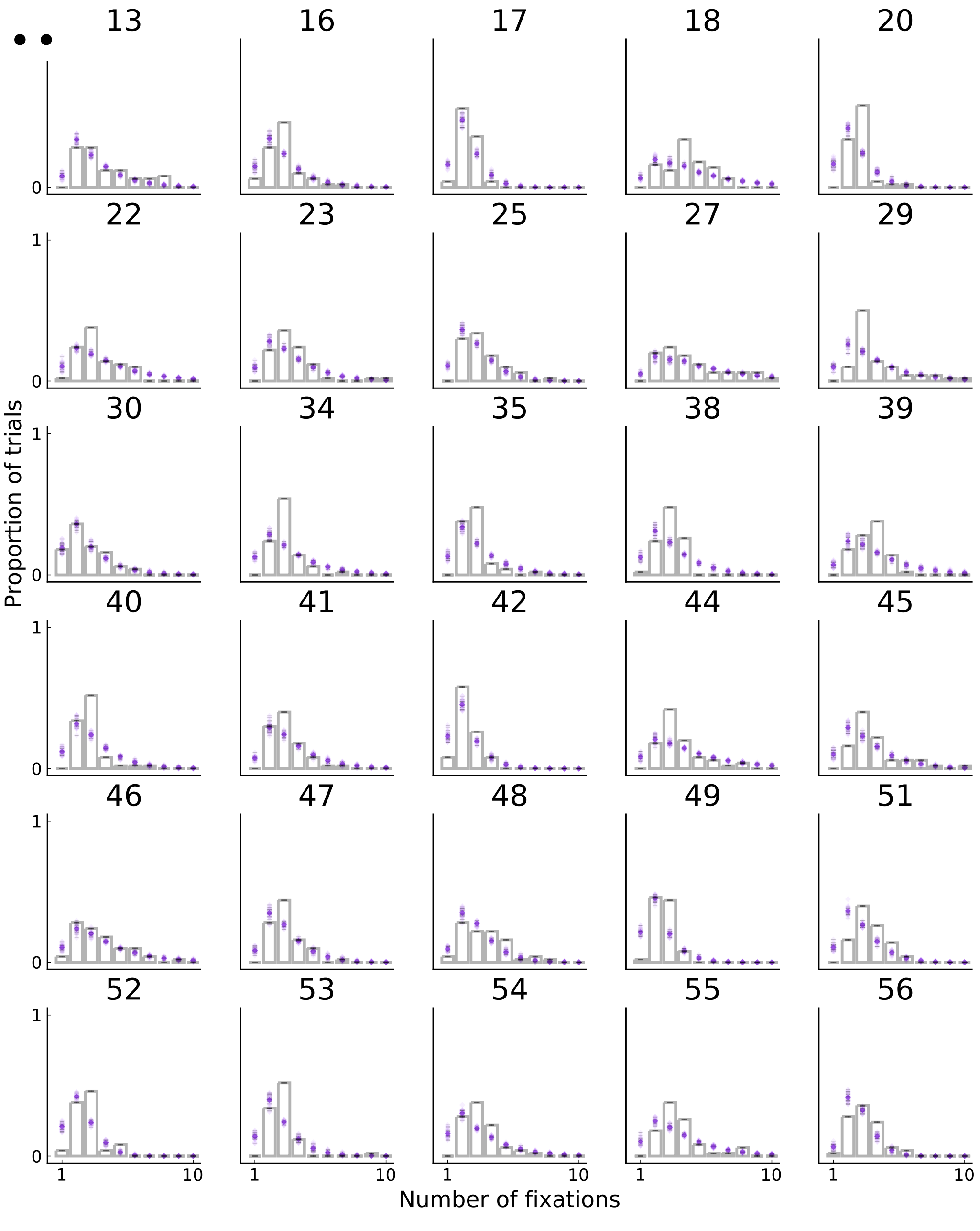

Figure I: Individual fits for binary dataset, Figure 4A: Histogram of number of fixations in a trial. Light purple shows predictions of the top 30 parameter configurations. Dark purple shows the aggregate prediction. Human data are in black and model predictions are in purple. See the caption of Figure A for details.

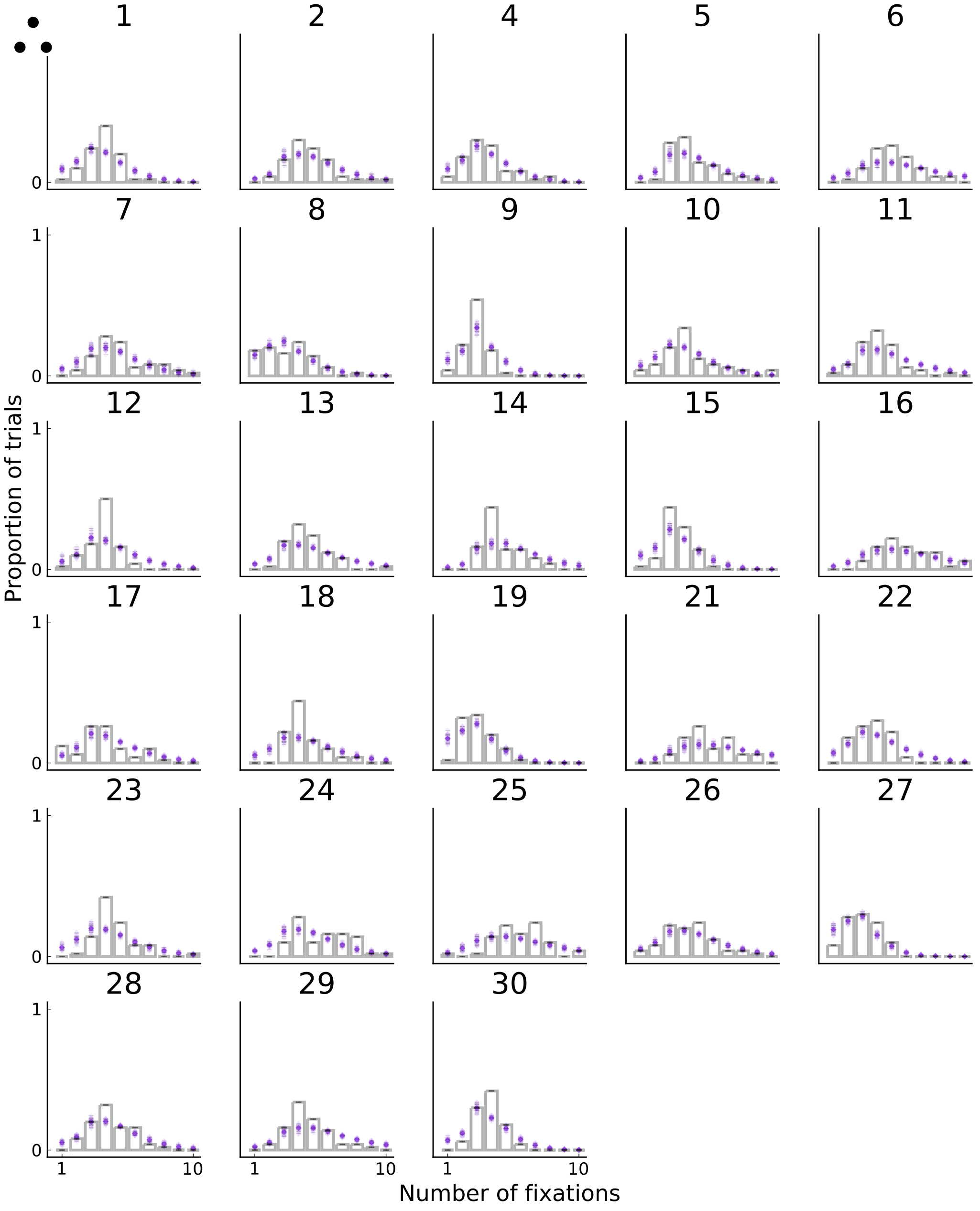

Figure J: Individual fits for trinary dataset, Figure 4A: Histogram of number of fixations in a trial. Light purple shows predictions of the top 30 parameter configurations. Dark purple shows the aggregate prediction. Human data are in black and model predictions are in purple. See the caption of Figure A for details.

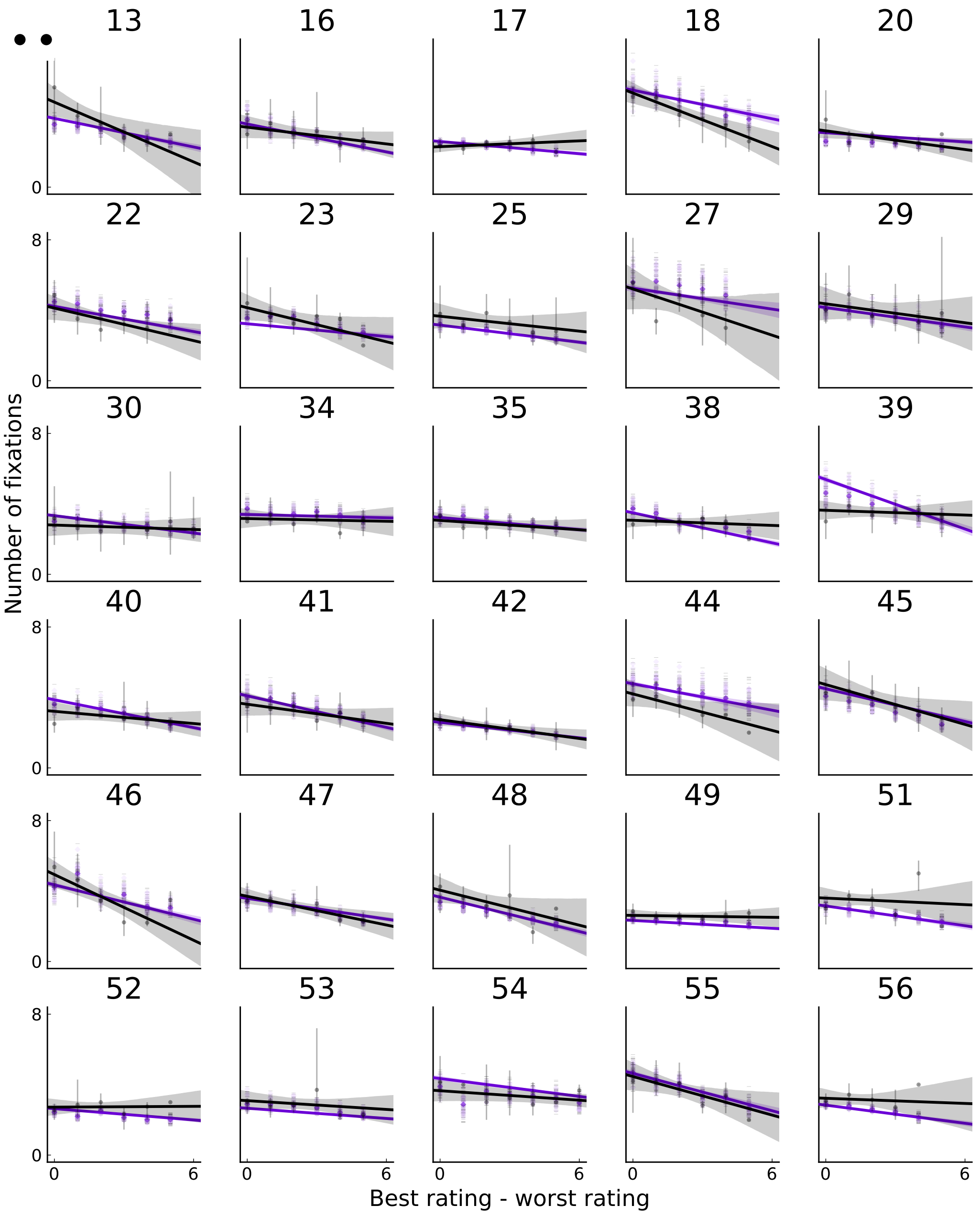

Figure K: Individual fits for binary dataset, Figure 4B: Number of fixations as a function of decision difficulty, as measured by the relative rating of the best item. Curves show the predictive mean and 95% confidence intervals of a Bayesian linear model. Human data are in black and model predictions are in purple. See the caption of Figure A for details.

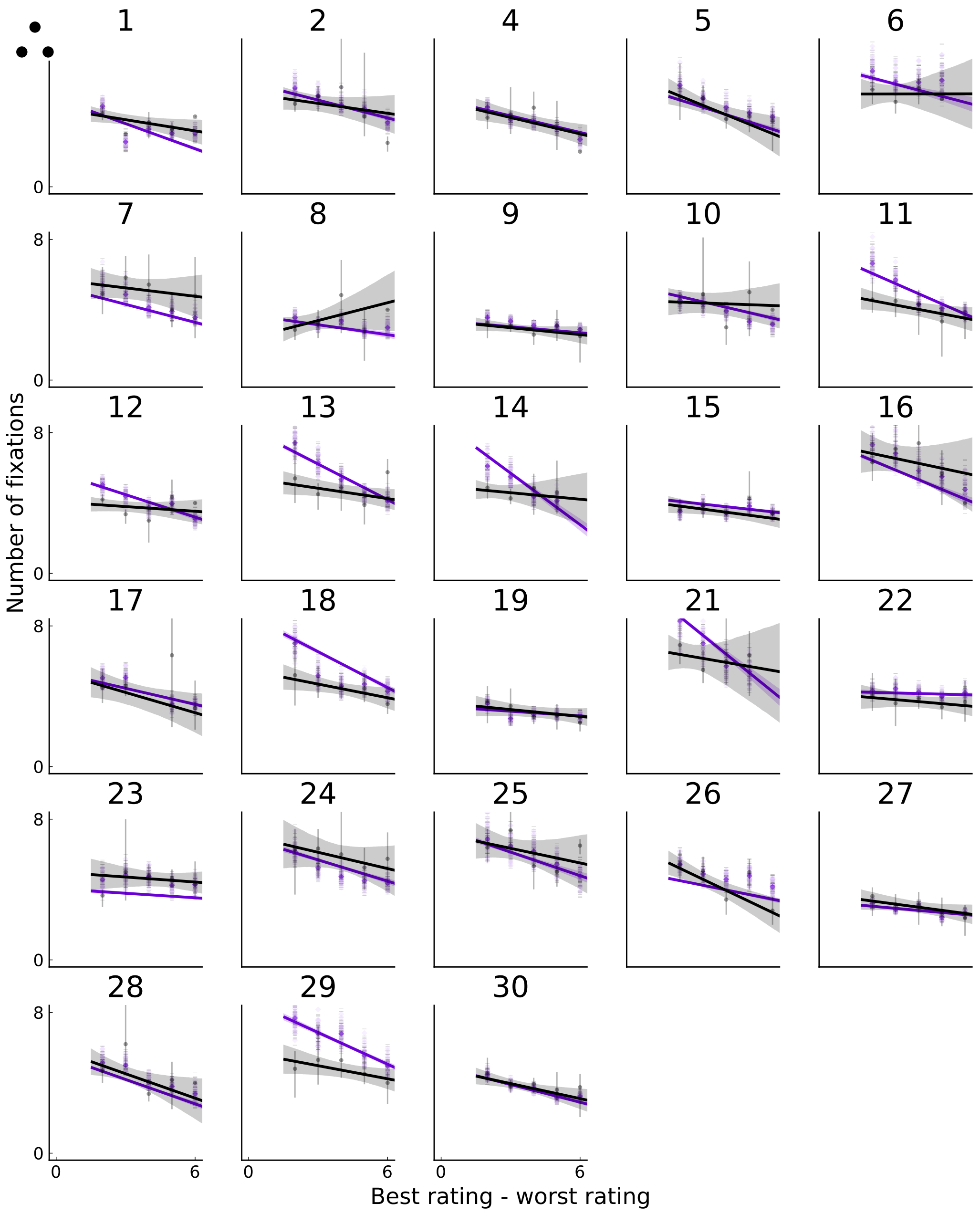

Figure L: Individual fits for trinary dataset, Figure 4B: Number of fixations as a function of decision difficulty, as measured by the relative rating of the best item. Curves show the predictive mean and 95% confidence intervals of a Bayesian linear model. Human data are in black and model predictions are in purple. See the caption of Figure A for details.

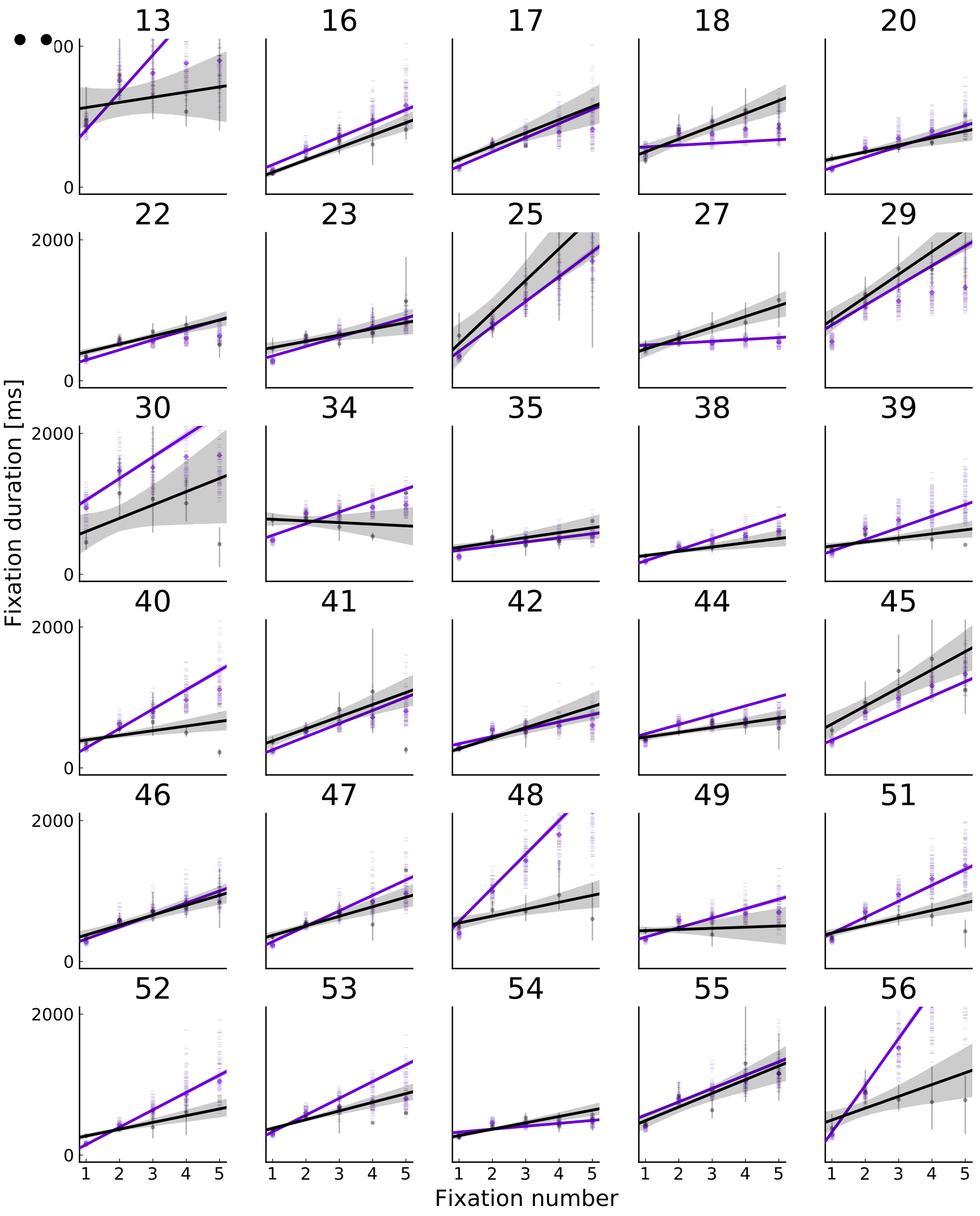

Figure M: Individual fits for binary dataset, Figure 4C: Duration of fixation by fixation number. Final fixations are excluded. Curves show the predictive mean and 95% confidence intervals of a Bayesian linear model. Human data are in black and model predictions are in purple. See the caption of Figure A for details.

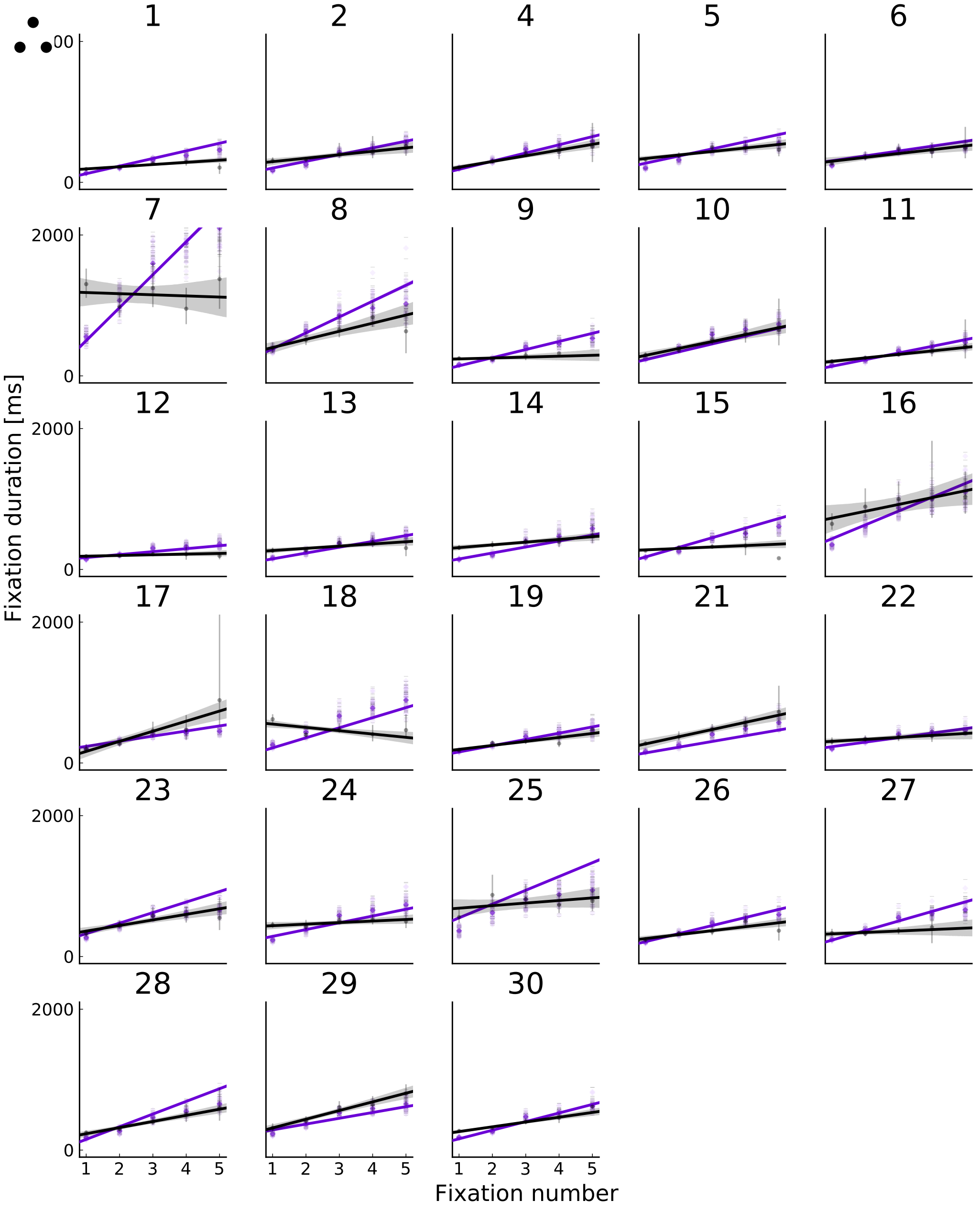

Figure N: Individual fits for trinary dataset, Figure 4C: Duration of fixation by fixation number. Final fixations are excluded. Curves show the predictive mean and 95% confidence intervals of a Bayesian linear model. Human data are in black and model predictions are in purple. See the caption of Figure A for details.

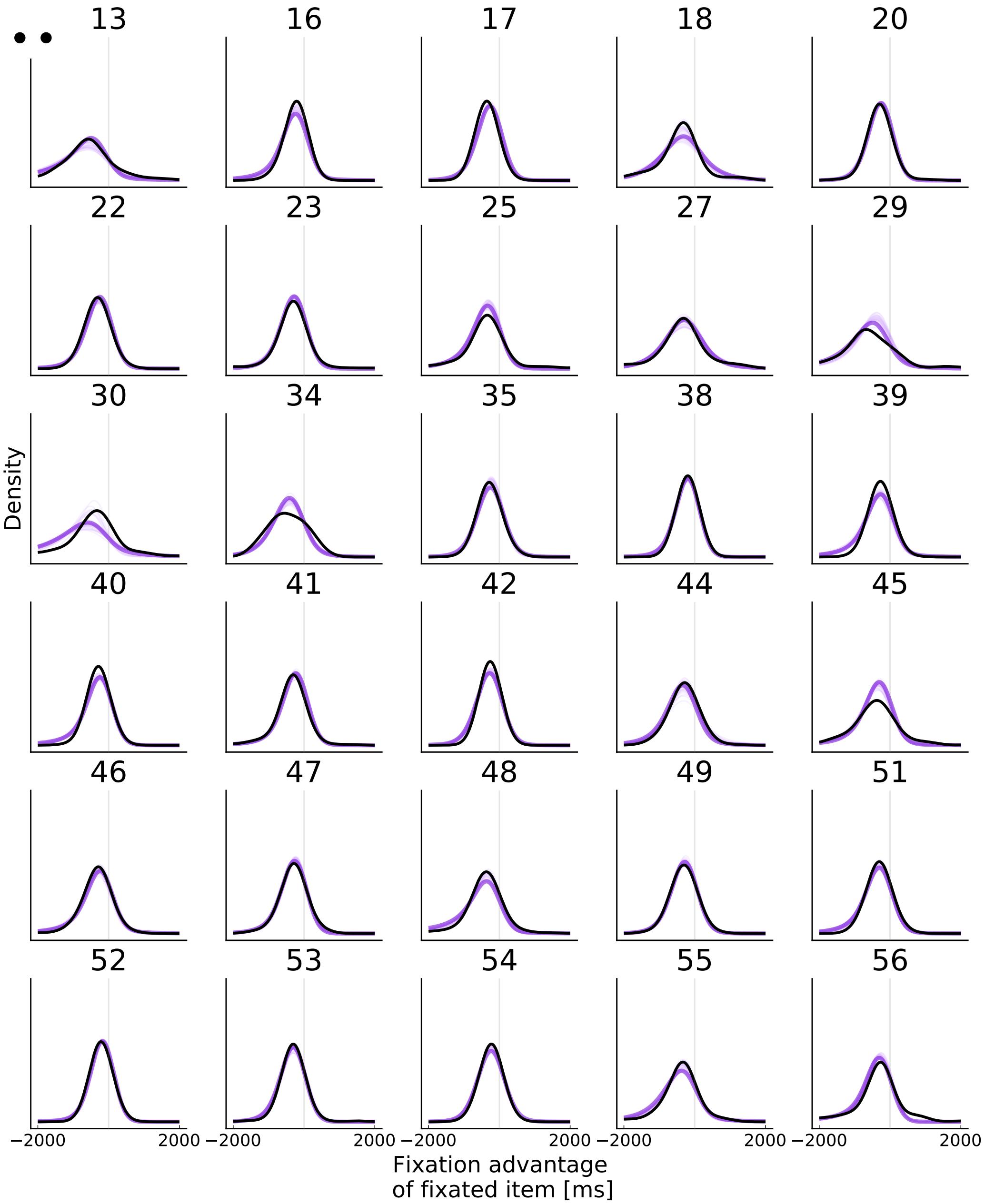

Figure O: Individual fits for binary dataset, Figure 5A: Distribution of fixation advantage of the fixated item, computed at the beginning of each new fixation. First fixations are excluded in this plot. Fixation advantage is defined as the cumulative fixation time to the item minus the mean cumulative fixation time to the other item(s). Light purple shows predictions of the top 30 parameter configurations. Dark purple shows the aggregate prediction. Human data are in black and model predictions are in purple. See the caption of Figure A for details.

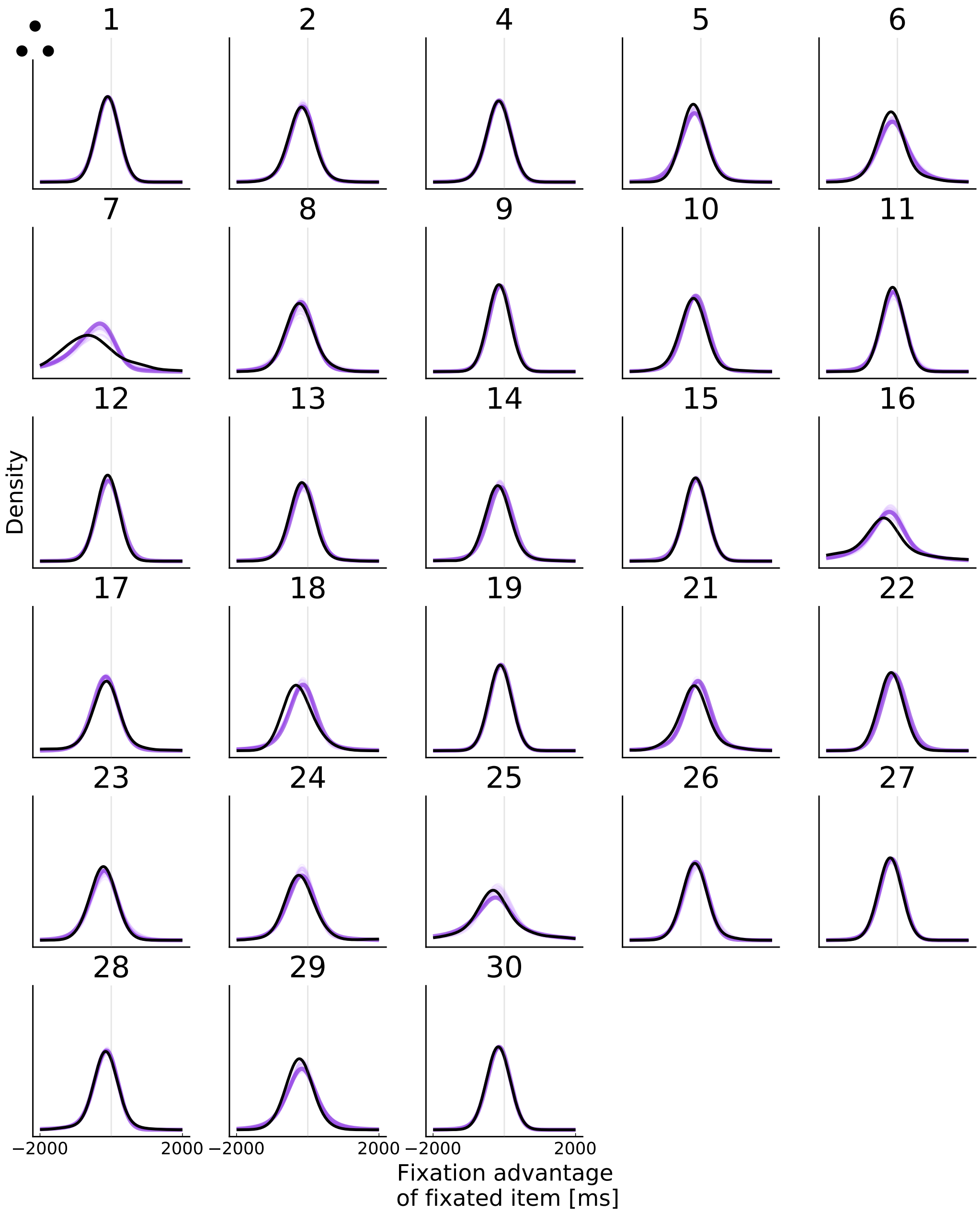

Figure P: Individual fits for trinary dataset, Figure 5A: Distribution of fixation advantage of the fixated item, computed at the beginning of each new fixation. First fixations are excluded in this plot. Fixation advantage is defined as the cumulative fixation time to the item minus the mean cumulative fixation time to the other item(s). Light purple shows predictions of the top 30 parameter configurations. Dark purple shows the aggregate prediction. Human data are in black and model predictions are in purple. See the caption of Figure A for details.

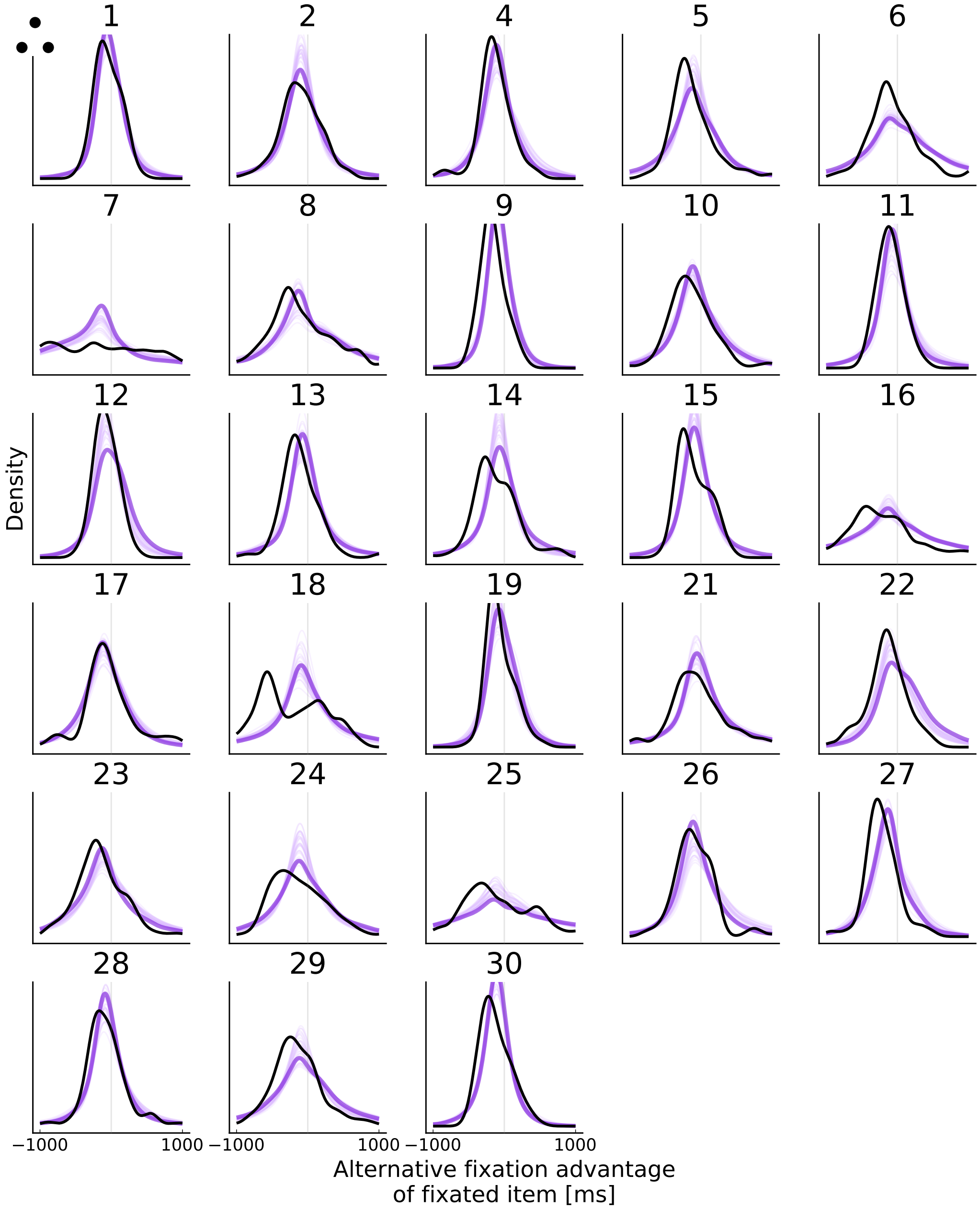

Figure Q: Individual fits for trinary dataset, Figure 5B: Distribution of fixation advantage of the fixated item versus the item item that could have been fixated but was not (i.e., the item that was not fixated on either this fixation nor the previous fixation). First and second fixations are excluded in this plot. Light purple shows predictions of the top 30 parameter configurations. Dark purple shows the aggregate prediction. Human data are in black and model predictions are in purple. See the caption of Figure A for details.

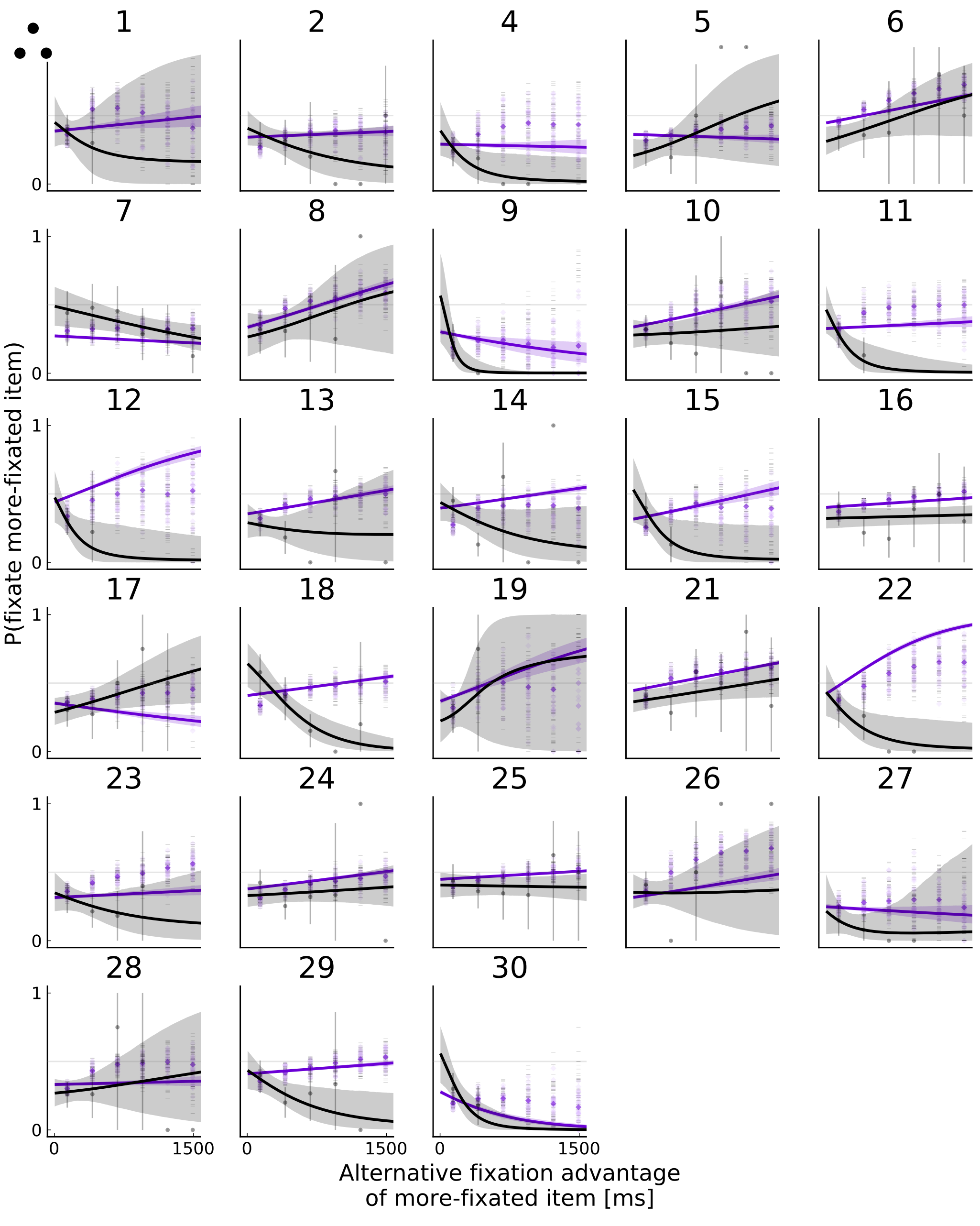

Figure R: Individual fits for trinary dataset, Figure 5C: The probability that the item with greater alternative fixation advantage is fixated as a function of that advantage. Curves show the predictive mean and 95% confidence intervals of a Bayesian linear model. Human data are in black and model predictions are in purple. See the caption of Figure A for details.

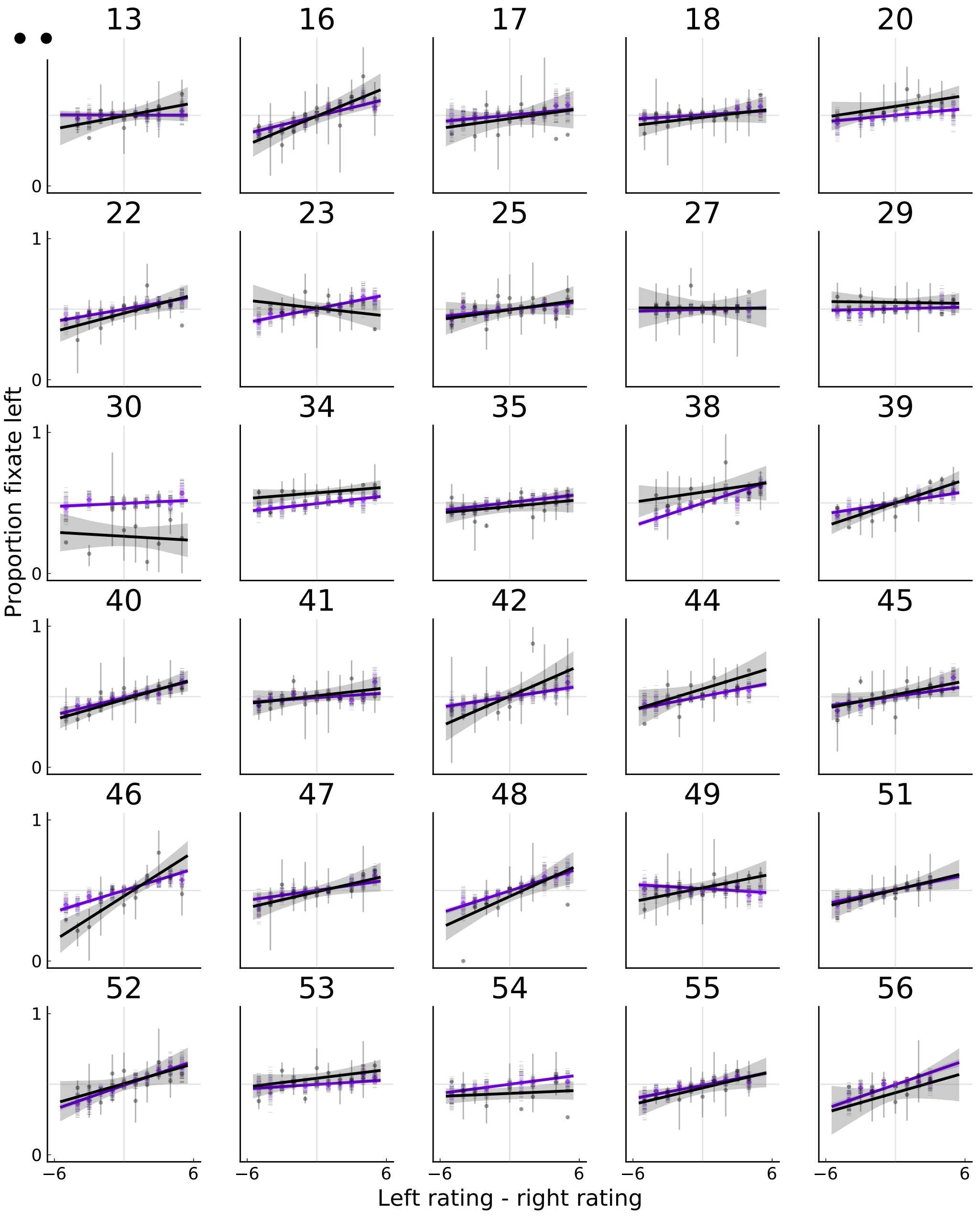

Figure S: Individual fits for binary dataset, Figure 6A: Proportion of time fixating the left item as a function of its relative rating. Note that the predicted effect in the binary case is due to a confound and not value-directed attention; see main text. Curves show the predictive mean and 95% confidence intervals of a Bayesian linear model. Human data are in black and model predictions are in purple. See the caption of Figure A for details.

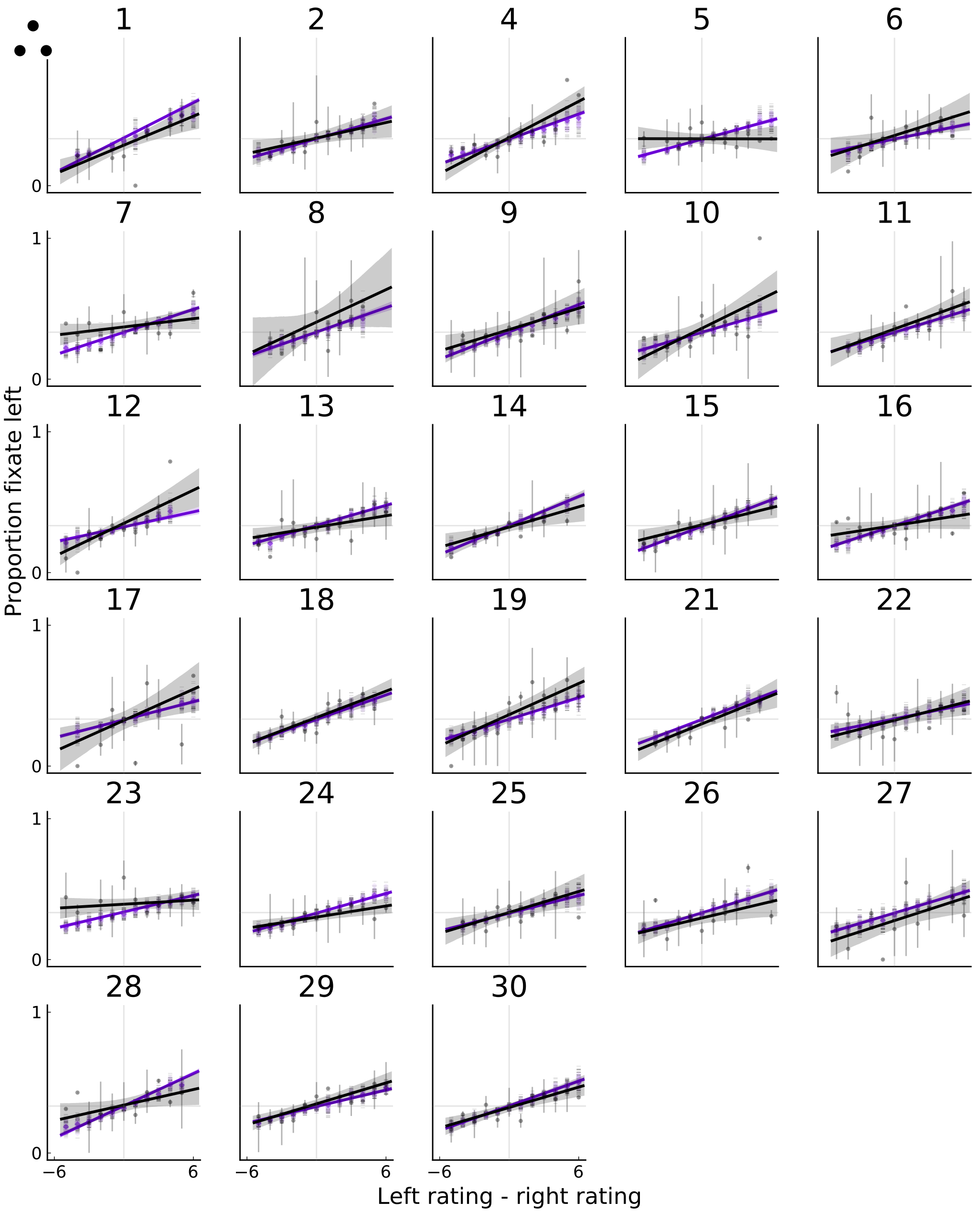

Figure T: Individual fits for trinary dataset, Figure 6A: Proportion of time fixating the left item as a function of its relative rating. Note that the predicted effect in the binary case is due to a confound and not value-directed attention; see main text. Curves show the predictive mean and 95% confidence intervals of a Bayesian linear model. Human data are in black and model predictions are in purple. See the caption of Figure A for details.

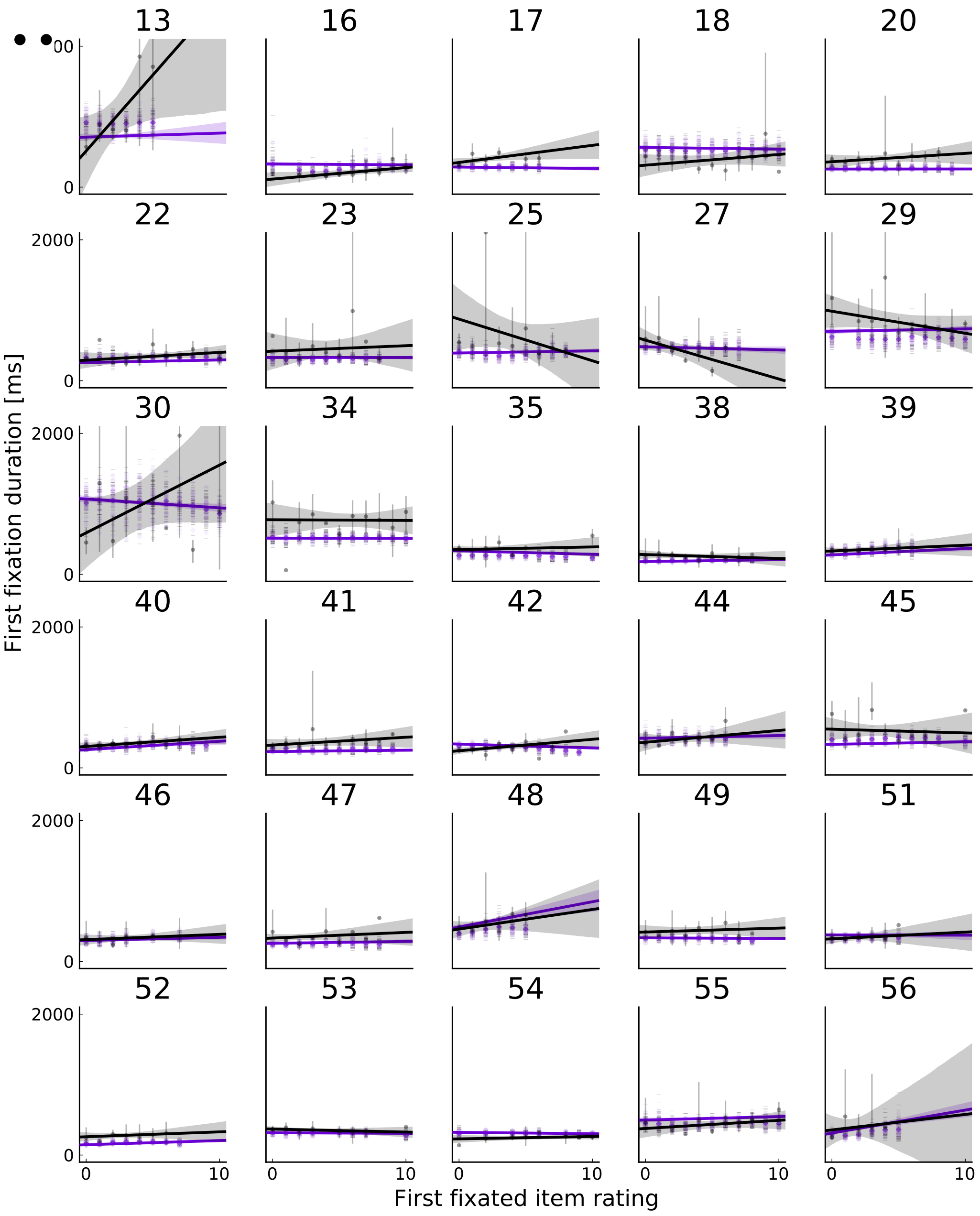

Figure U: Individual fits for binary dataset, Figure 6B: First fixation duration as a function of the rating of the first-fixated item. Curves show the predictive mean and 95% confidence intervals of a Bayesian linear model. Human data are in black and model predictions are in purple. See the caption of Figure A for details.

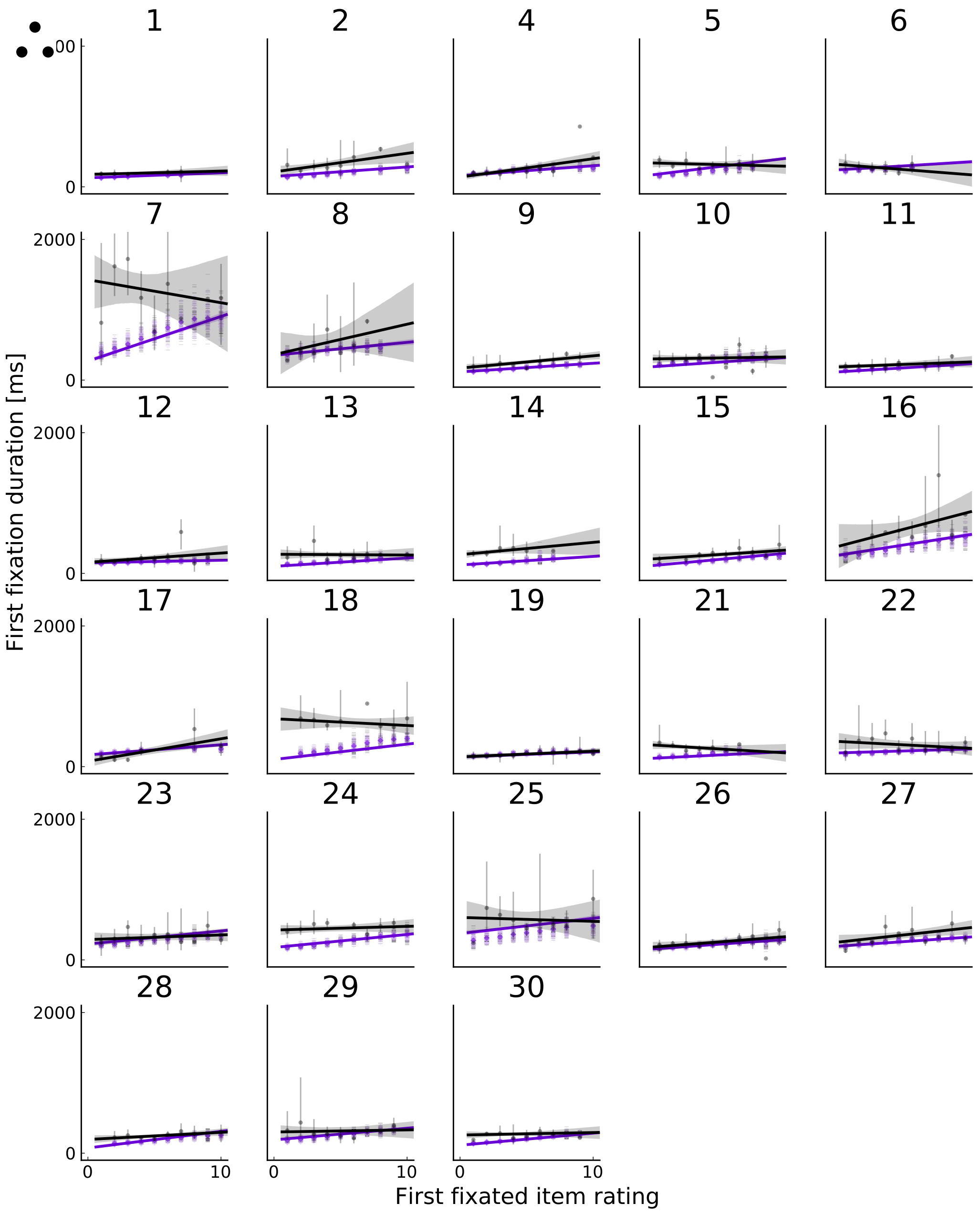

Figure V: Individual fits for trinary dataset, Figure 6B: First fixation duration as a function of the rating of the first-fixated item. Curves show the predictive mean and 95% confidence intervals of a Bayesian linear model. Human data are in black and model predictions are in purple. See the caption of Figure A for details.

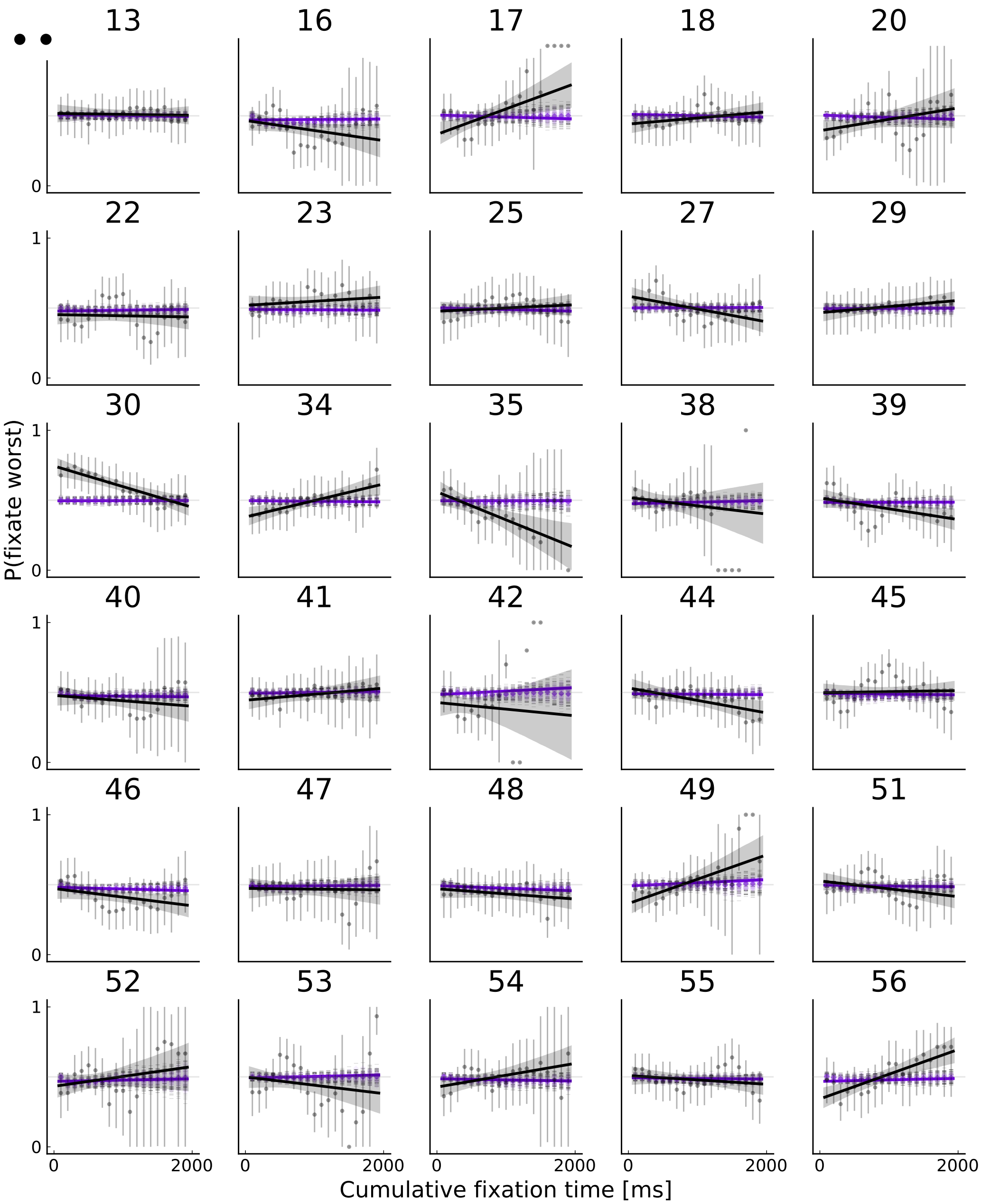

Figure W: Individual fits for binary dataset, Figure 6C: Probability of fixating the lowest rated item as a function of the cumulative fixation time to any of the items. Curves show the predictive mean and 95% confidence intervals of a Bayesian linear model. Human data are in black and model predictions are in purple. See the caption of Figure A for details.

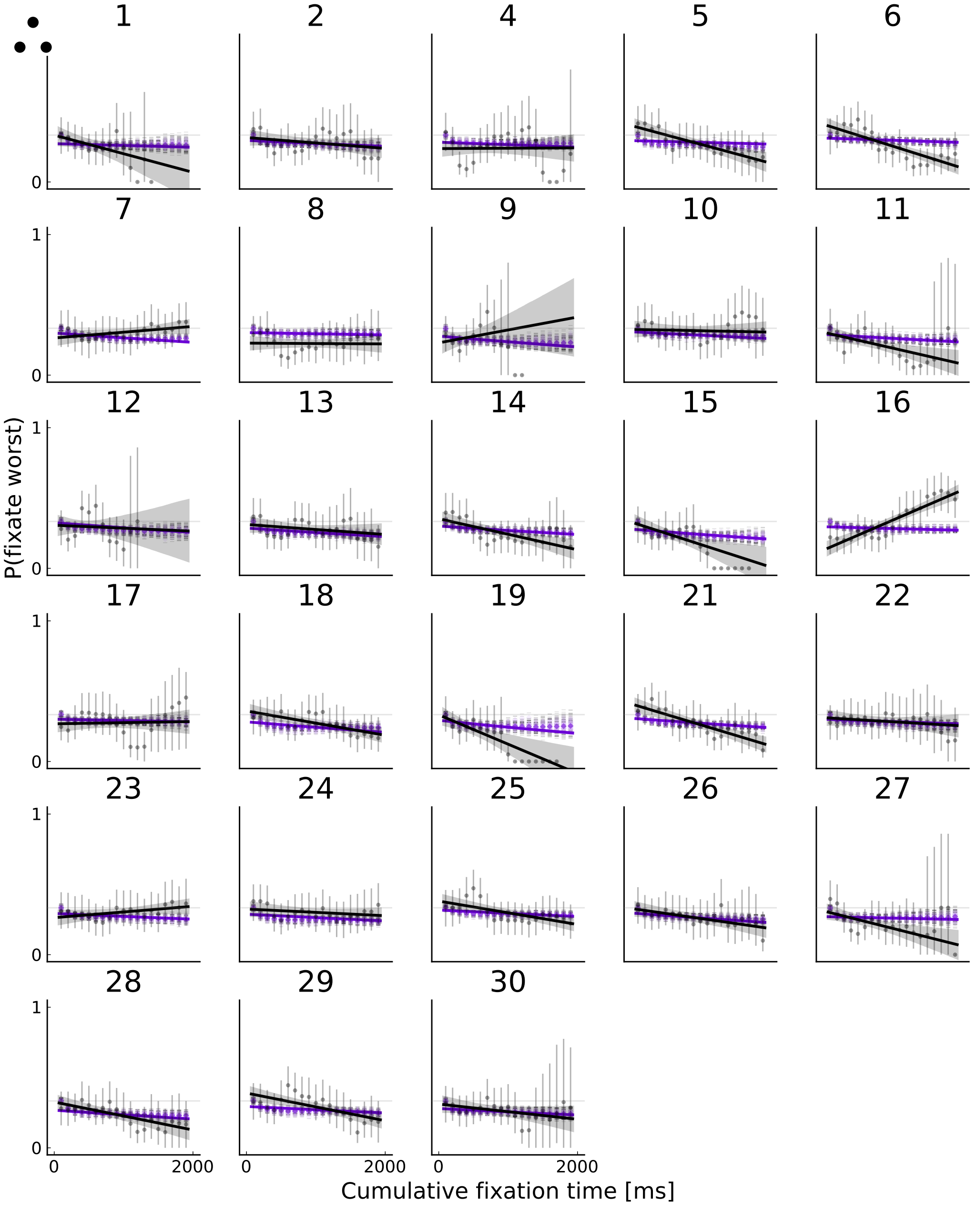

Figure X: Individual fits for trinary dataset, Figure 6C: Probability of fixating the lowest rated item as a function of the cumulative fixation time to any of the items. Curves show the predictive mean and 95% confidence intervals of a Bayesian linear model. Human data are in black and model predictions are in purple. See the caption of Figure A for details.

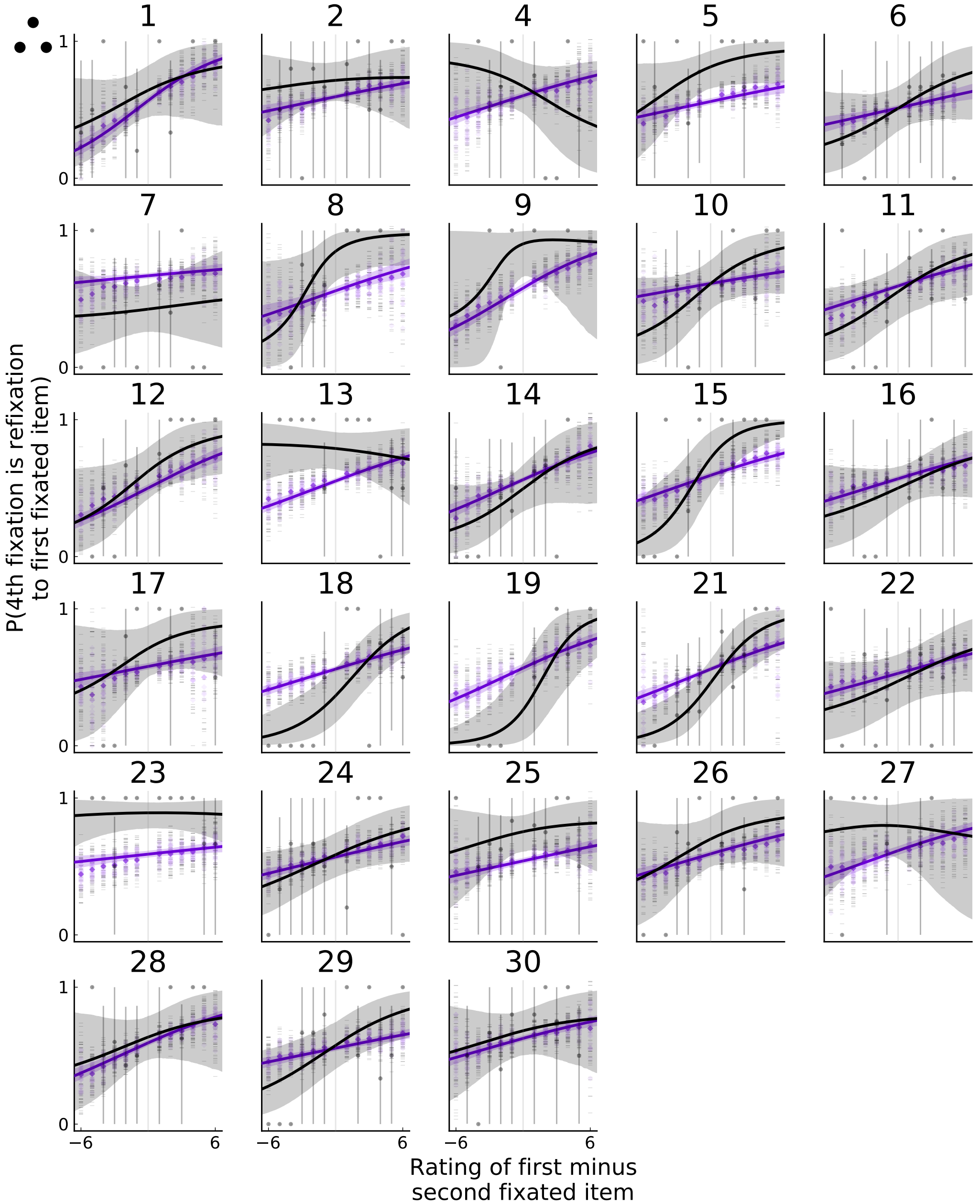

Figure Y: Individual fits for trinary dataset, Figure 6D: Probability that the fourth fixation is to the first-fixated item as a function of the difference in rating between that item and the second-fixated item. Curves show the predictive mean and 95% confidence intervals of a Bayesian linear model. Human data are in black and model predictions are in purple. See the caption of Figure A for details.

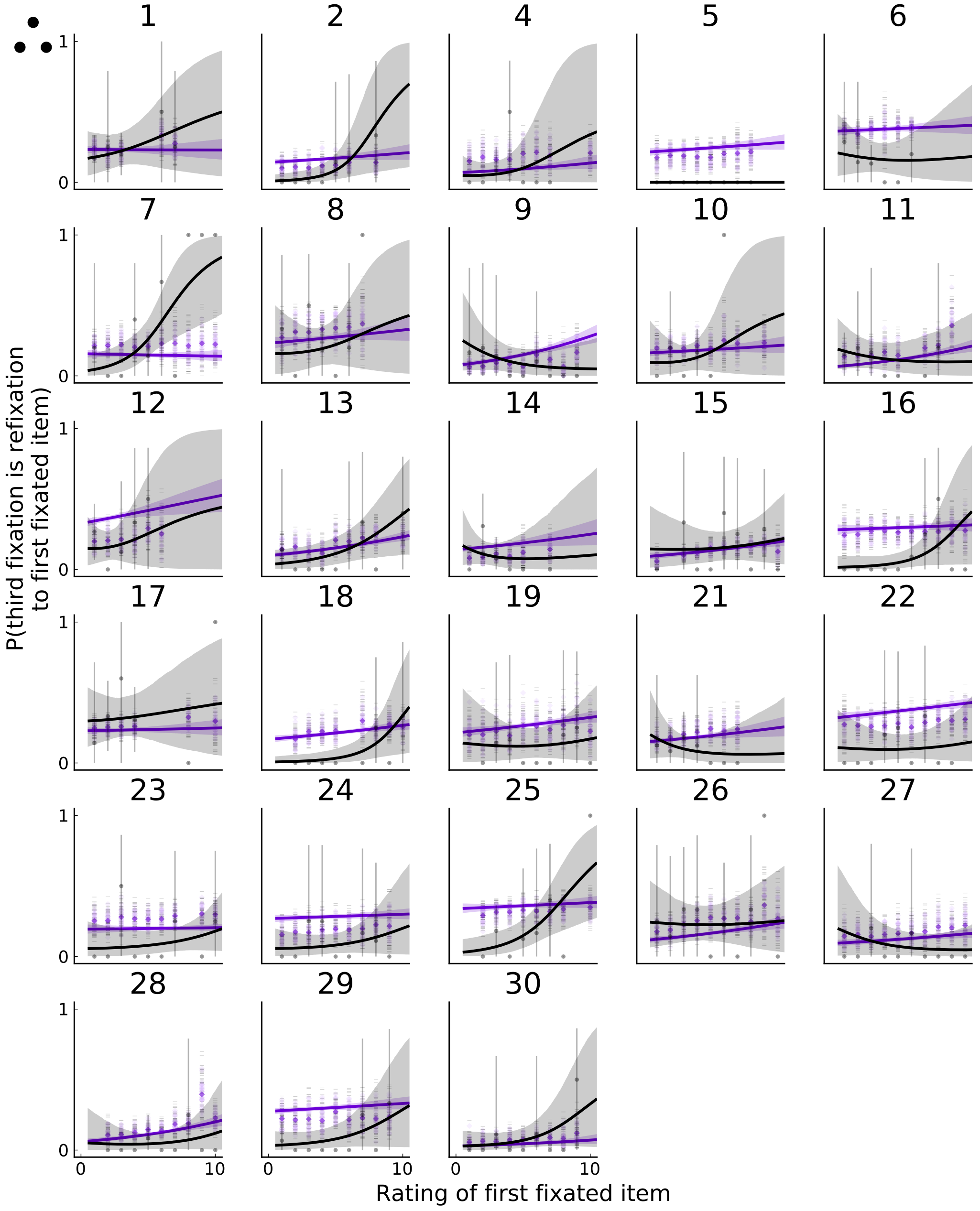

Figure Z: Individual fits for trinary dataset, Figure 6E: Probability that the third fixation is to the first fixated item as a function of its rating. Curves show the predictive mean and 95% confidence intervals of a Bayesian linear model. Human data are in black and model predictions are in purple. See the caption of Figure A for details.

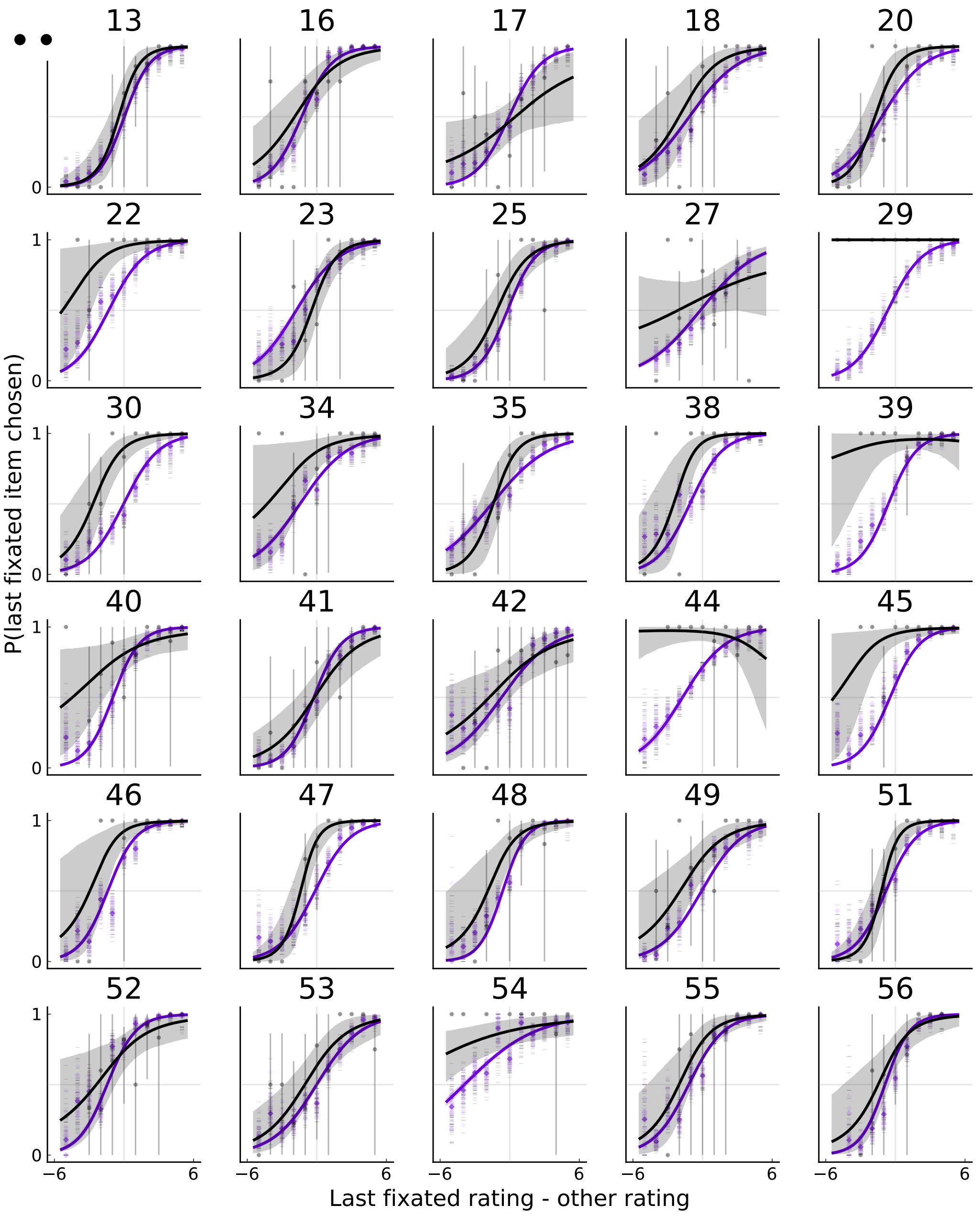

Figure : Individual fits for binary dataset, Figure 7A: Probability that the last fixated item is chosen as a function of its relative rating. Curves show the predictive mean and 95% confidence intervals of a Bayesian linear model. Human data are in black and model predictions are in purple. See the caption of Figure A for details.

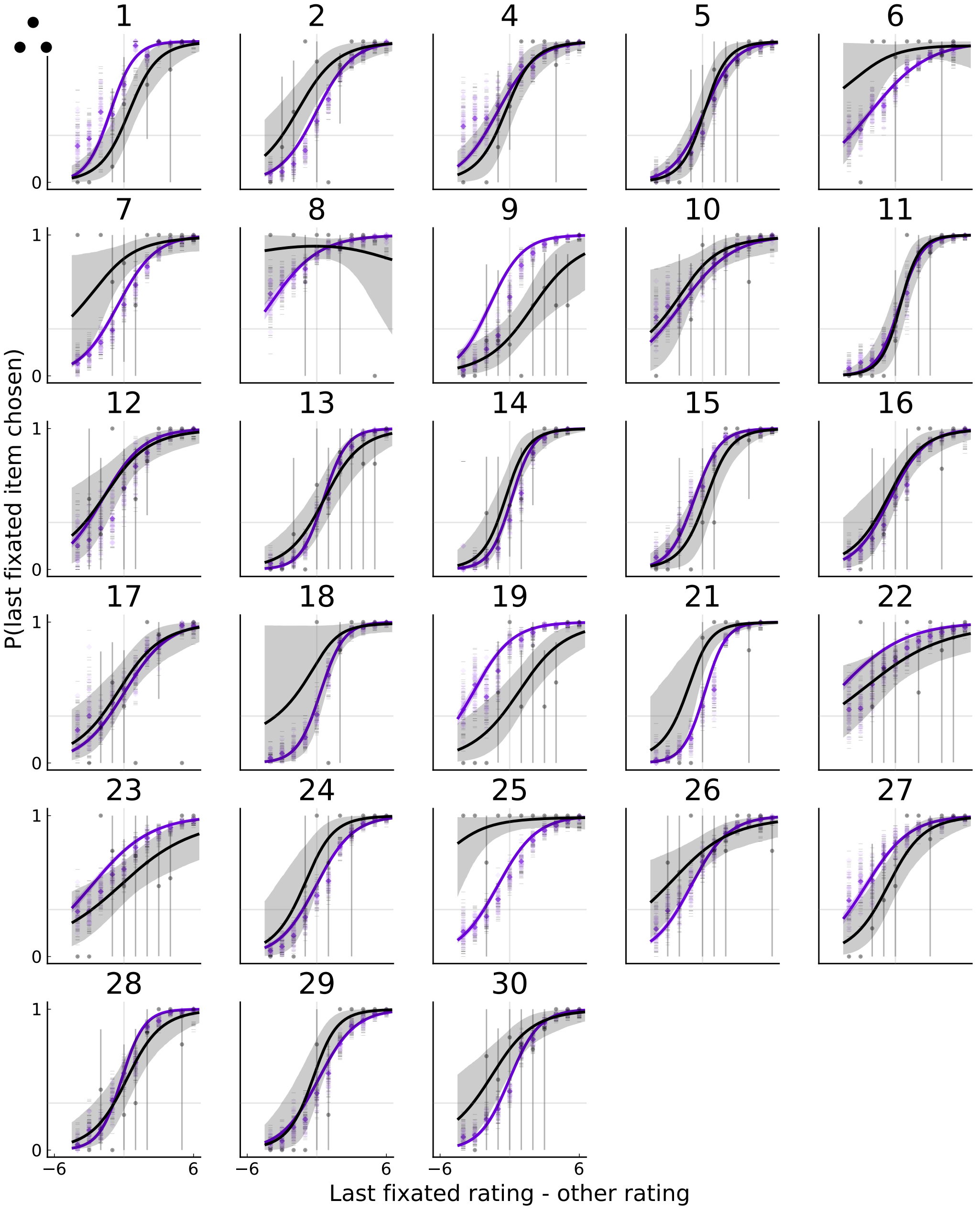

Figure : Individual fits for trinary dataset, Figure 7A: Probability that the last fixated item is chosen as a function of its relative rating. Curves show the predictive mean and 95% confidence intervals of a Bayesian linear model. Human data are in black and model predictions are in purple. See the caption of Figure A for details.

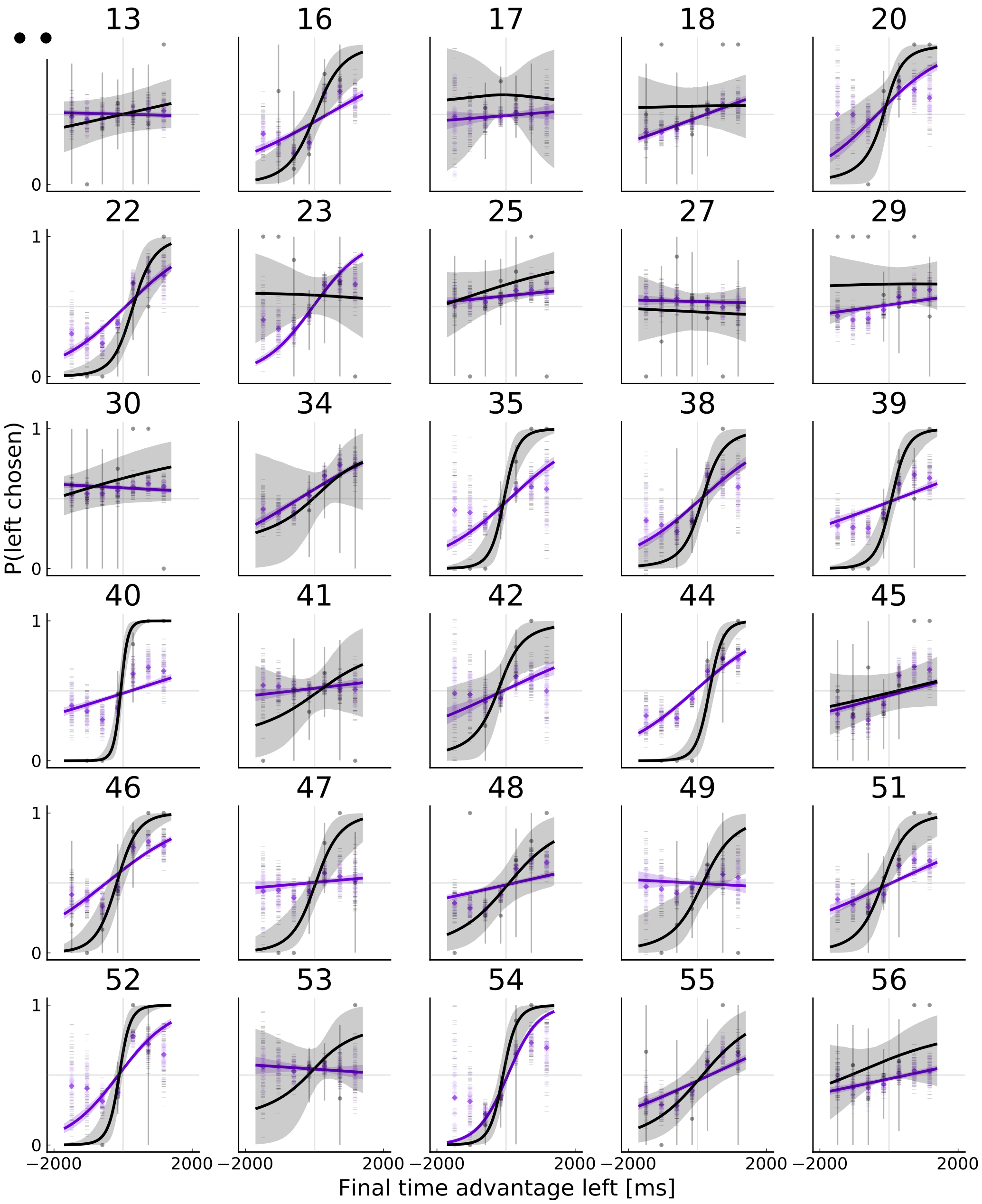

Figure : Individual fits for binary dataset, Figure 7B: Probability that the left item is chosen as a function of its final fixation advantage, given by total fixation time to the left item minus the mean total fixation time to the other item(s). Curves show the predictive mean and 95% confidence intervals of a Bayesian linear model. Human data are in black and model predictions are in purple. See the caption of Figure A for details.

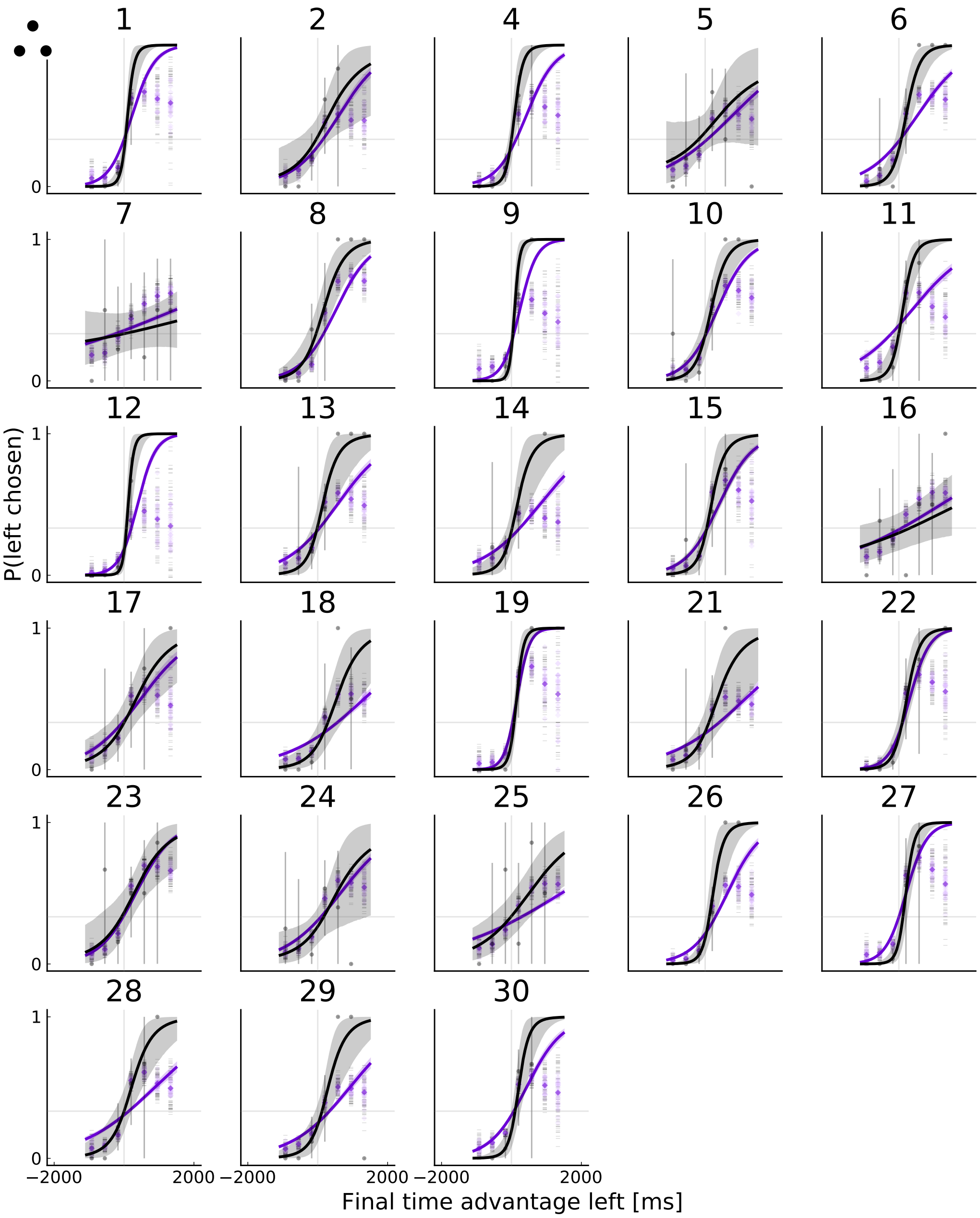

Figure : Individual fits for trinary dataset, Figure 7B: Probability that the left item is chosen as a function of its final fixation advantage, given by total fixation time to the left item minus the mean total fixation time to the other item(s). Curves show the predictive mean and 95% confidence intervals of a Bayesian linear model. Human data are in black and model predictions are in purple. See the caption of Figure A for details.

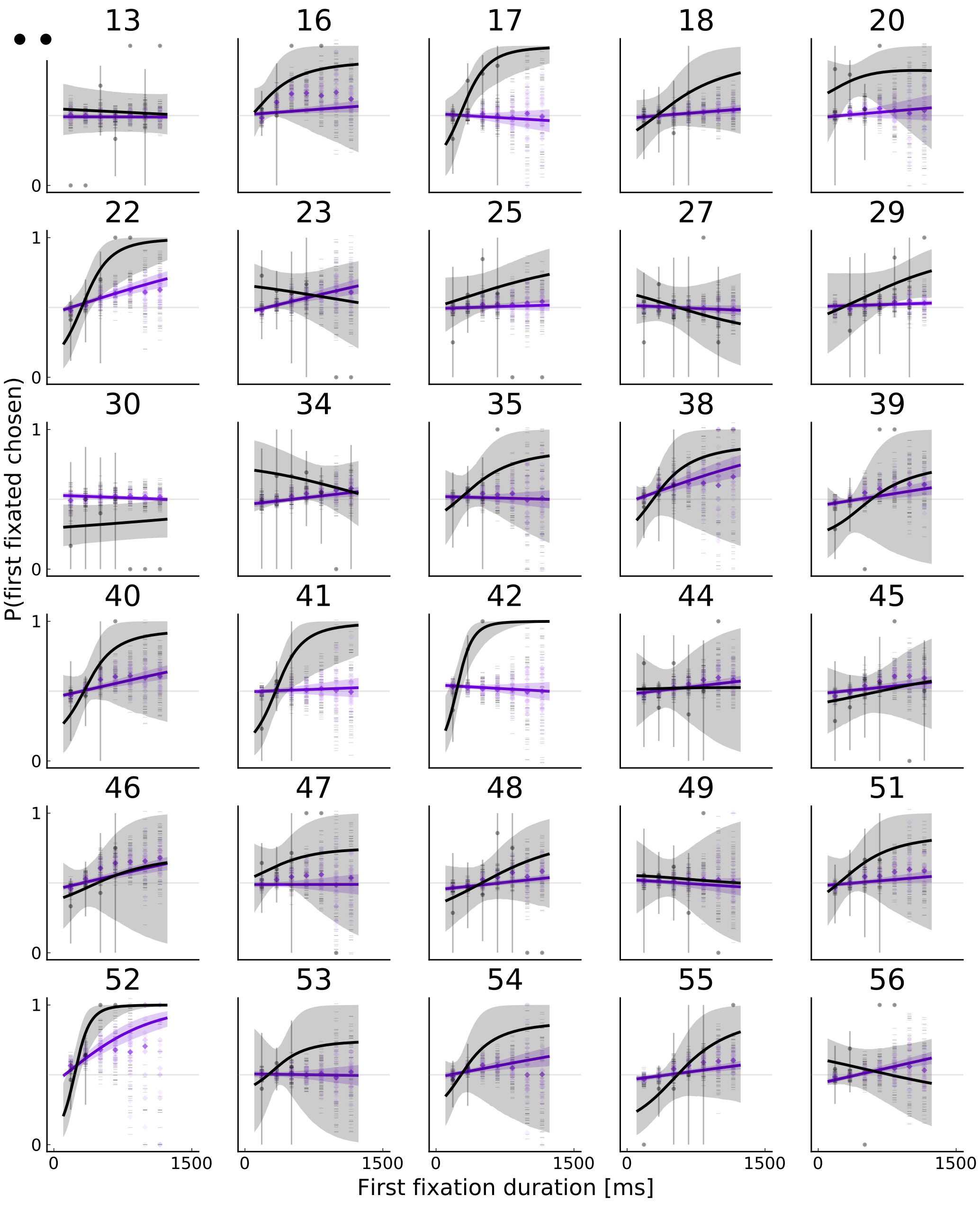

Figure : Individual fits for binary dataset, Figure 7C: Probability of choosing the first-seen item as a function of the first-fixation duration. Curves show the predictive mean and 95% confidence intervals of a Bayesian linear model. Human data are in black and model predictions are in purple. See the caption of Figure A for details.

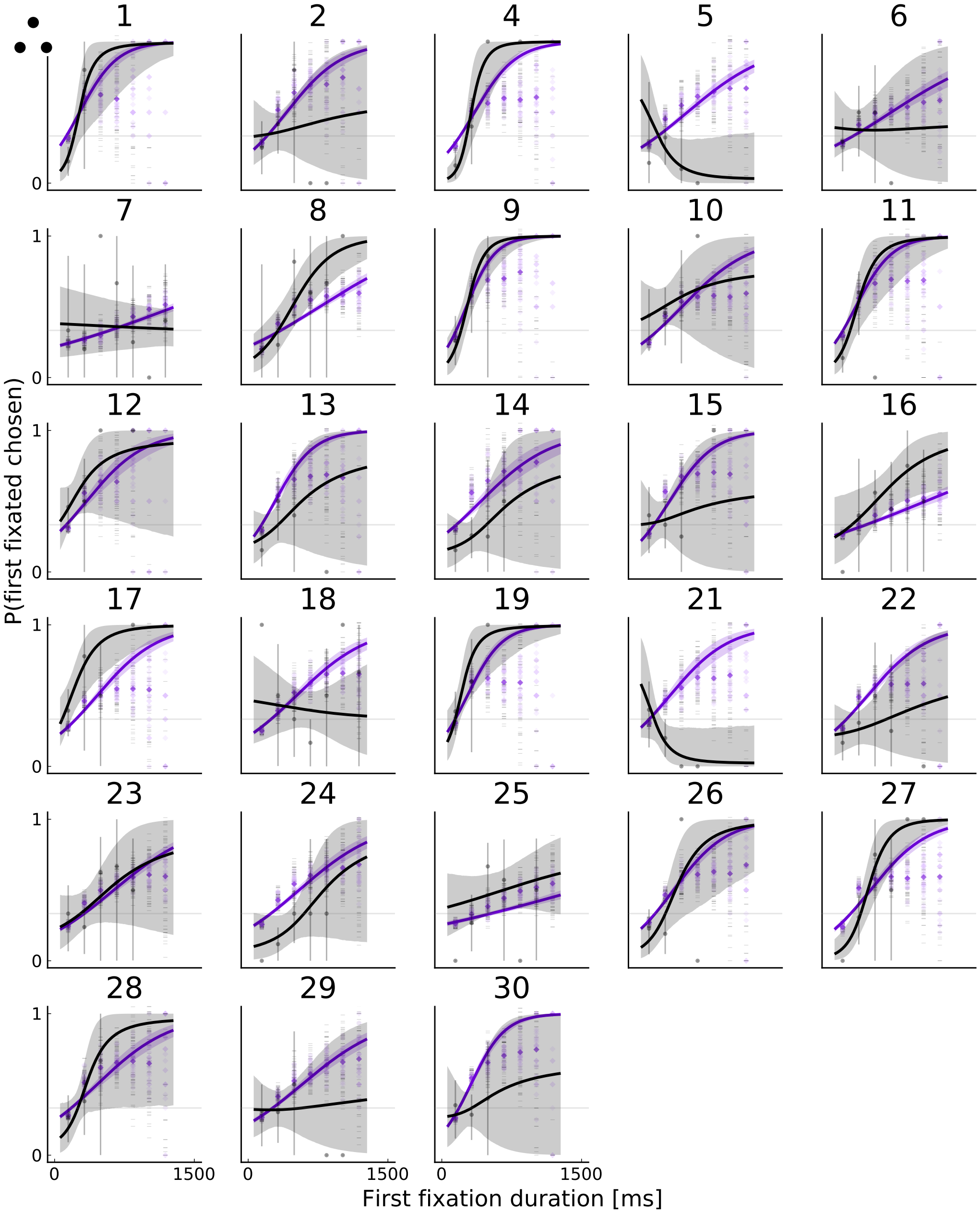

Figure : Individual fits for trinary dataset, Figure 7C: Probability of choosing the first-seen item as a function of the first-fixation duration. Curves show the predictive mean and 95% confidence intervals of a Bayesian linear model. Human data are in black and model predictions are in purple. See the caption of Figure A for details.
