# Supplementary material for: Fixation patterns in simple choice reflect optimal information sampling
Source: PLoS Comput Biol. 2021 Mar 26;17(3):e1008863. doi: 10.1371/journal.pcbi.1008863 (PMC8026028; doi:10.1371/journal.pcbi.1008863)
Supplement: Attachment — Submitted filename: plos-response.pdf [file pcbi.1008863.s003.pdf]

Dear Dr. Gershman,

Please find enclosed our manuscript "Fixation patterns in simple choice reflect optimal information sampling" which we are resubmitting for consideration for publication in *PLoS Computational Biology* (manuscript number PCOMPBIOL-D-20-01568).

We thank you and the reviewers for the insightful comments on the previous version of this manuscript. The reviews highlighted several important concerns about the model assumptions. Fortunately, we believe we were able to address all of them, and that in doing so we think we have improved the paper. We hope that you and the reviewers will agree that the manuscript is now ready for publication.

To briefly summarize the key changes:

- We now consider the possibility that the decision maker's prior beliefs are biased with respect to the true distribution of item values in the experiment. By fitting the degree of prior bias to the data, we find that the main qualitative mismatch to the behavioral data (not predicting choice biases in the binary case) is largely eliminated.
- We plot the predictions of the aDDM alongside our model predictions in all the plots. Overall, we find that our model performs similarly to the aDDM, despite the fact that the aDDM samples fixation locations and durations directly from the empirical distribution.
- We demonstrate the robustness of our parameter estimation method by showing that we can accurately recover parameters when applying the estimation method to model simulations.
- We include information on fits to individual participant data in the SI and show that the model performs well and key phenomena are reproduced at the individual level.
- We plot model predictions for the "overall value" effect and discuss how the model speaks to the additive vs. multiplicative debate.
- Throughout the text, we have added material and refined existing material to improve clarity.

In the following pages, we address the specific concerns raised in each review.

Note that in addition to the updated manuscript, we include a version with tracked changes. For your convenience, we have also created a draft with inline figures, which can be found at the URL below. The text is exactly the same as the officially submitted manuscript, and the line numbers match as well.

Draft with inline figures: http://fredcallaway.com/pdfs/callaway-plos-attention.pdf

We look forward to your response!

Best,

Fred Callaway, Antonio Rangel, Tom Griffiths

Responses to R1

We thank the reviewer for the careful reading of the paper and the useful comments. As you will see, we have carried out a substantial additional number of analyses that we hope have further strengthened the paper.

For the reviewer's convenience, we have copied below the original comments in regular font-type, and added our responses in bold. Note also that a draft with inline figures (and matching line numbers) is available here:
http://fredcallaway.com/pdfs/callaway-plos-attention.pdf

COMMENTS AND RESPONSES

In the manuscript entitled "Fixation patterns in simple choice reflect optimal information sampling", Callaway and colleagues propose a model for optimal information sampling in value-based decision making. The model is fitted to binary and trinary choices data, and data fit and misfits are discussed.

Overall, I found this paper quite interesting. It raises an important issue about the optimality of fixations in value-based decision making that has not been explored so far. However, I am concerned about the theoretical assumptions of such a model.

Main points

1. My first concern is about the concept of information sampling in value-based decision making and its dependency on visual inputs. While visual inputs constitute the accumulated evidence in perceptual decision making (as described in the sequential sampling framework), the evidence accumulated in value-based decisions has less to do with visual aspects of the stimuli (as they are typically easy to identify based on their appearances) and more with values retrieved from memory. In fact, value based decisions can also be carried out without any visual input: One could ask participants to press left if they prefer a Kit-kat and right if they prefer a Bounty, without presenting Kit-kats and Bounties on the left and right sides of the screen. Participants could also be asked to choose between a right and a left option and to fixate a cross in the middle of the screen. Therefore, fixations should not be seen as instrumental or necessary for value based-decisions. Because of this, the authors' position, i.e., that "fixations are deployed to sample information optimally in order to make the best possible choice" [line 39] is to me inappropriate in value-based decisions. The authors say that such an account comes from perceptual decision making, where fixations are instrumental for the accumulation of evidence (the more attention to a specific area of the visual field, the better discriminating evidence I can gather). This is however not the case for value-based decisions, as it is possible to gather information for or against one option without looking at it (because the evidence is stored in memory).

Thank you for pushing us to address and clarify this important point.

We agree that in value-based decision making, the value samples driving the choice are largely generated from internal information, based on the properties of the stimuli that are shown to the decision maker. However, this does not mean that fixations do not matter. As an extreme example, consider a supermarket shelf with hundreds of items. Since the decision maker cannot evaluate all the items in parallel (i.e., simultaneously), fixations matter because they guide attention towards a small number of items at a time for which value samples can be drawn with most accuracy. That is, visual attention (indicated by fixations) helps to guide internal attention. This is the key assumption of models like the aDDM, and the key assumption in our model also.

One important question is whether value samples can be drawn from items that are not being fixated. The model currently makes the simplifying assumption that samples are drawn only when an item is attended, but one can easily imagine extensions of the model in which attention facilitate value sampling (say, by increasing the sampling rate or decreasing its noise), but in which samples can be also be drawn from non-fixated items (albeit at a slower rate or with more noise).

Surprisingly, the model does quite well in accounting for the data despite making this simplifying assumption. However, we hope that the paper motivates further work exploring the role of this specific assumption.

We have made two changes to the paper to address this.

In the Introduction, we briefly mention the type of sampling we have in mind (emphasis added)

> Given that such problems are characterized by both a different objective function (maximizing a scalar value rather than accuracy) and a different source of information (e.g. sampling from memory [41–43] rather than from a noisy visual stimulus), it is far from clear that optimal information sampling models will still provide a good account of fixations in this setting.

In the Discussion, we address this point more fully:

Optimal information sampling models may appear inappropriate for value-based decision- making problems, in which perceptual uncertainty about the identity of the different choice items (often highly familiar junk foods) is likely resolved long before a choice is made. Two features of the model ameliorate this concern. First,the samples underlying value-based decisions are not taken from the external display (as in perceptual decisions), but are instead generated internally, perhaps by some combination of mental simulation and memory recall [41–43]. Second, the model makes the eye-mind assumption [62, 63]: what a person is looking at is a good indicator of what they are thinking about. Importantly, these assumptions implicitly underlie all sequential sampling models of value-based decision-making.

2. The other, related, theoretical concern has to do with the use of "selective/sequential", as opposed to "parallel" information sampling. The authors say that, in the DDM, "the same amount of information about all options" is received "in parallel" [line 60], and the DDM constitutes the optimal algorithm for that kind of situation. However, in the aDDM, "decisions are based on sequentially accumulated value samples" [line 31] and that "the optimal algorithm when the decision maker can sample information selectively is unknown" [line 62]. In their model, participants get samples only from the item they are gazing at, and can only get samples from the other item(s) by switching the gaze towards them, which is costly. I have 3 points related this:

First, a clarification on the term "parallel": we did not intend to distinguish between sequential and parallel in this context (in most contexts, that is of course the natural comparison). All evidence accumulation models are sequential in the sense that samples are taken over time (not all at once). By "parallel" we meant that information about all items is acquired at the same rate (i.e., every instant a sample is taken from all available options), and thus there is no need to sequentially allocate attention between them (i.e., to choose at any instant which option to sample from. We have replaced "parallel" with "at the same rate" to hopefully improve clarity on this point.

(a) can the author actually provide evidence that participants are indeed gathering information selectively/sequentially? This would mean to find evidence that people only gather information about an option at a time. This seems to be a crucial point: If there is actually no evidence for this, then the DDM is actually still the optimal algorithm (well, at least for 2-options tasks).

We assume that the reviewer is referring here to whether samples are drawn at the same rate for every option (i.e., so that at every instant a sample is drawn for every available option) or instead, sampling is influenced by fixation locations.

The reviewer is correct that in the former case, the optimal model is the DDM, and that a key assumption of our model is that fixations affect sampling. This assumption is motivated and justified by previous studies showing that visual

attention affects choices, including the aDDM and related literatures cited in the paper (quoted below). We have additionally added citations to two reviews that summarize the extensive evidence that visual attention affects value-based decision making.

> Second, visual attention affects the decision-making process. In particular, items that are fixated longer are more likely to be chosen [6–13], unless they are aversive, in which case they are chosen less frequently [7, 14]. These findings have been explained by the Attentional Drift Diffusion Model (aDDM), in which the value samples of the fixated item are over-weighted relative to those of unfixated ones [9, 10, 12, 13]. **See refs 63 and 50 for reviews.**

This previous work includes the two datasets we model. In particular, Figure 7 shows three of the main effects that have been used to support the theory that attention affects the sampling process, and therefore that information is not gathered at the same rate for all items. Although, in principle, there are other ways to explain these effects, we note that (1) the explanation of selective sampling provided by the aDDM is widely accepted in the field and (2) our model, which assumes selective sampling, provides a good account of many patterns in the fixation data.

(b) The other point is that there should be some clarification about mutual inhibition. In both the DDM and aDDM, evidence is accumulated as a single sum, and there is perfect inhibition between the 2 options. This means that the higher the evidence in favour of one option at a time point t (including the noise), the more discounted the evidence for the other option will be. As far as I understand (please correct me if I am wrong), in their account, it seems like there is no inhibition at all. So I think what the authors are proposing here is not only a model sequential information sampling, but also information sampling in which the evidence accumulated for one option is independent from the one accumulated from the other. I think it should be an open question whether this is optimal in the case of sequential information sampling as well. In case I misunderstood this point, I would still like the question of inhibition to be clarified in the paper, explaining how that plays a crucial role in optimality.

This paper investigates an optimal sampling model of fixations and simple choice, not its algorithmic implementation in the brain. The issue of inhibition is critical in algorithmic implementations, but not in the optimal Bayesian analysis. Following Tajima et al. (2016, 2019), we assume that the samples are drawn from a Gaussian with mean equal to the corresponding item's value. Given this assumption, the integration of evidence follows exact Bayesian inference. Adding inhibition would necessarily violate the rational norm of Bayesian inference, and thus does not seem appropriate for our model given our emphasis on optimality.

One further clarification. In the traditional two-alternative choice DDM (and the original aDDM) the role of inhibition depends on the exact neural network used to implement or approximate the algorithm. In one form of the algorithm, there is no *inhibition* so much as there is one accumulator that tracks the difference in value between the two items (This is distinct from e.g. the leaky competing accumulator model which, posits two separate accumulators that inhibit each other). Interestingly, for binary choice, the DDM in which one accumulator represents the difference in value is equivalent to tracking two separate estimates of each item's value as shown by Tajima et al., (2016).

(c) I think it is incorrect to say that in the aDDM samples are accumulated sequentially. In the aDDM, the drift-rate changes within a trial to account for attentional fluctuations. However, the drift-rate is the same as in the DDM: it assumes that evidence is accumulated in a single sum and with perfect inhibition, so never only for one option alone.

We apologize again for the confusing language. Here we do not mean to contrast sequential with parallel (see discussion about our confusing usage of "parallel" above). Instead, we simply mean that at each timestep a sample is drawn (either one for each item or one for the difference between items) and that these samples accumulated over time. For this reason, DDMs are sometimes referred to as "sequential sampling models." We hope that removing the term parallel from the text clears up this confusion.

3. In a previous paper (Smith & Krajbich, Psychol Sci, 2018) the aDDM was compared with a model in which "gaze merely adds evidence, providing a fixed advantage for the attended option", also called by the authors an additive model (Krajbich, Current Opinion in Psychology, 2018). The authors found that only the aDDM could account for overall-value effects. Can the author explain what their optimal model say about such effects?

Thank you for bringing up this important effect, which we had neglected in the previous draft. We now include the overall value effect in Figure 3. Our model predicts a strong overall value effect because Bayesian updating implies a multiplicative effect of attention (as does the aDDM). In particular, the expected estimated value after N samples of an item is:

$$\mathbf{E[\mu] = \mu_0 + [(N * \sigma_2^{-2}) / (\lambda_0 + N * \sigma_2^{-2})] * (true\_value - \mu_0)}$$

That is, the expected prior mean is a hyperbolic function of the amount of attention with start and end point determined by the prior mean and the true value (Armel & Rangel 2008).

Also can the authors discuss the similarities between theirs and this additive model accounts?

Yes! We have added the following section in the discussion:

> Our results also suggest another (not mutually exclusive) mechanism by which the attention- choice correlation can emerge: value-directed attention. We found that the optimal model with no prior bias (α = 1) predicts an attention-choice correlation in the trinary choice case. This is because, controlling for true values, an increase in estimated value (e.g., due to sample noise) makes the model more likely to both fixate and choose an item. This could potentially help to resolve the debate over additive vs. multiplicative effects of attention on choice [11, 13]. While the prior-bias mechanism predicts a multiplicative effect, the value-directed attention mechanism predicts that fixation time and choice will be directly related (as predicted by the additive model). Although we did not see strong evidence for value-directed attention in the binary dataset, such a bias has been shown in explicit information gathering settings [65] and could be at work in other binary choice settings.

We emphasize, however, that our model does *not* include an additive causal effect of attention on choice. Instead, our model posits a direct relationship between attention and choice (not moderated by value) due to a common cause; they are both driven by estimated value. Thus, although our model may predict similar behavioral patterns to the additive model, the underlying assumptions of the models are quite different.

4. I think it would benefit to report the aDDM fits (quantitative and qualitative) in this paper as well, even though they might be found in the original papers. These could work well as a benchmark model and make very clear where the authors' proposed account and the aDDM converge/diverge.

Excellent idea! We now include predictions of the aDDM with the parameters estimated in the original papers in all the plots. In the SI, we replicate several key figures from the 2010 and 2011 papers to demonstrate correct implementation.

Please note that the aDDM samples fixations from the empirical distribution, rather than predicting them as in our model. Thus, these models cannot be compared in their ability to predict properties of the fixation sequences (especially Figures 5 and 6).

We have made changes throughout the paper to address this point.

5. In my opinion, the authors should provide a better definition of optimality in value-based decision making. Can their view be better grounded in existing literature perhaps?

The criterion for optimality in models of value-based decision making that do not take into account any constraints on the process of choice is maximizing expected utility. This criterion follows from the standard treatment of statistical decision theory (Savage, 1954, von Neumann, 1944). Our approach

extends this criterion to the case where an agent needs to allocate a costly attentional resource that trades off with the utility of the outcomes.

We have added a paragraph to the introduction to clarify how we define optimality:

> In this paper, we draw these threads together by defining a model of optimal information sampling in canonical simple choice tasks and investigating the extent to which it accounts for fixation patterns and their relation to choices. In a value-based choice, optimal information sampling requires maximizing the difference between the value of the chosen item and the cost of acquiring the information needed to make the choice. Our model thus falls into a broad class of models that extend classical rational models of economic choice [26, 27] to additionally account for constraints imposed by limited cognitive resources [28–33]. However, as is common in this approach, we stop short of specifying a full algorithmic model of simple choice. Instead, we ask to what extent people's fixations are consistent with optimal information sampling, without specifying how the brain actually implements an optimal sampling policy.

6. I found the Methods a bit brief and at the same time quite hard to read. I suggest to make them a bit more accessible. I have a few points here:

Thank you for the feedback! We have expanded the methods to improve clarity.

(a) The model is fit at a group level, across two tasks, and only group-level patterns are shown for the model fits. Can the authors justify this choice and also show how individual fits actually look like? As said before, perhaps comparing their model to the aDDM?

We focused on group-level fits because we were especially interested in the ability of the model to *predict* differences between binary and trinary decisions (e.g. Figures 4C, 6A-C). By using one set of parameters for both datasets, we ensure that any differences in the predictions are *ex ante* consequences of the structure of the model (in particular, the assumption of optimal information sampling) rather than aspects of the data that the model captures *ex post* using different parameters.

However, in response to comments from you and R2, we have also performed individual fits and include versions of all the figures in the paper with one panel for each participant in the Supplementary Information.

(b) Can the authors provide evidence that the model is identifiable and that the parameters (in this case at the group level) are unbiased? Is the use of the chosen trial summary statistics for fitting sufficient and are they unbiased?

Excellent suggestion!

We have carried out an extensive validation of the parameter estimation methods, which is now presented in the Supplementary Information.

As described there, we achieve fairly accurate recovery (Pearson r between 0.92 and 0.98 for all parameters except the inverse temperature β for which r = 0.83). We do see slight bias, with the worst bias of -0.087 occurring (appropriately) for the prior bias term, α, which takes values between 0 and 1. Note that the summary statistics are certainly not sufficient in the technical sense; however, this is almost always the case in practice (see Sunnaker et al. 2013 [89]).

Minor points

- Figure 3B: Better use quantiles and not kernel density estimators to compare RT/fixation time distributions.

Good suggestion! We now plot quantiles in addition to the KDE for all fixation time distributions.

- Response times (versus reaction), as we are in the value-based and not perceptual domain.

Both terms are commonly used in the literature. We hope the current implementation is clear, although we are sympathetic that the distinction raised by the reviewer has merit.

- I believe the (more) correct name is diffusion decision model (and not drift diffusion model), see for example: Ratcliff, R., Smith, P. L., Brown, S. D., & McKoon, G. (2016). Diffusion decision model: Current issues and history. Trends in cognitive sciences, 20(4), 260-281.

We have made this change.

- Repetition of "process", line 29.

- "choice" is missing, line 81.

- Something funny on line 512.

- Quantiles spelled wrong, line 533.

We intended "quintile"(as opposed to quartile or decile). To clarify, this sentence now reads "...binned into quintiles (i.e., five bins containing equal numbers of data points)".

Responses to R2

We thank the reviewer for the careful reading of the paper and the useful comments. As you will see, we have carried out a substantial additional number of analyses that we hope have further strengthened the paper.

For the reviewer's convenience, we have copied below the original comments in regular font-type, and added our responses in bold. Note also that a draft with inline figures (and matching line numbers) is available here:
http://fredcallaway.com/pdfs/callaway-plos-attention.pdf

COMMENTS AND RESPONSES

The manuscript presents an "optimal" joint model for choices, fixations, and fixation times in binary and ternary value-based decision making. The model is an optimal model in the sense that it assumes that an agent attempts to maximize their payoff while choosing from which of the options to sample (i.e., fixate on) which increases the information of the value of the option and when to stop and pick the option with the highest value. The model has only four free parameter (or five parameters, see below), but is able to predict an impressive number of empirical patterns and only fails to explain a few, especially for the binary choices. Importantly, the model performance is evaluated using 50% of hold-out data that was not used to fit the model.

Evaluation

There is a lot to like about this paper: It starts with an interesting perspective of the task of the participant, considers several data types (i.e., eye movements and choices), implements the idea in a formally and technically sophisticated manner, uses different data for model estimation and model testing, and the model provides an overall pretty good performance. Thus, on the positive side, the paper provides an impressive computational effort to value-based decision making. However, there are also a few negative issues, listed below, that somewhat limit the impact of the paper. The big lingering question is what we really learn from the model about the task? Thus, my main suggestions are about the framing of the results. Nevertheless, I believe these issues can be fixed in a revision by providing a more balanced view of the contribution of the model. In addition, there are a few situation which I believe some parts could be clarified a bit.

Main issues

1. This might be more of a reviewer than an author issue, but I have some problems with the usage of the term "optimal" in this manuscript. To me, the term "optimal model" implies that the model captures the relevant constraints of the task and implements the one strategy (or "metalevel" strategy in the present case) that, if followed, maximises the payoff. However, in the current case we now have the surprising situation that the data is largely consistent with the

optimal model for the three item case, but shows some more pronounced misfit for the two item case. What does this mean? One possibility is that participants do not follow the optimal strategy in the two item case, but they do follow it in the three item case. I find this possibility hard to believe. What would be the reason that when going from two to three items the behaviour of the participants changes so substantially?

The other possibility seems to be, and this is also the possibility explicitly mentioned by the authors in the discussion (ll. 345), that the optimal model is not really the optimal model and other optimal models could capture the pattern for both two and three items. But if this is the case, in what sense is then the original model actually optimal? In other words, what is the benefit of avoiding proposing a "specific algorithmic model" if the optimal model is treated in exactly the same manner as such an algorithmic model, its assumption are subject to change if they do not fit the data (for a discussion see e.g., Tauber et al., 2017, Psychological Review).

Several things in response to this important comment:

First, the goal of the exercise is to derive the optimal model of fixations and choice, subject to the informational constraints and costs specified by the model, and then test the extent to which the observed human behavior matches the optimal policy.

Second, optimality is defined with respect to the model, not with respect to the observed behavior. In other words, as emphasized in behavioral economics, the extent to which people use optimally the information at hand is a testable hypothesis.

Third, as the reviewer suggests at various points, the optimal model might fail to match the observed behavior, not because people act sub-optimally, but because the model of the underlying informational structure and costs is incorrect.

Fourth, and most importantly, the failure to explain some key patterns in the binary choice data was due to the treatment of the prior beliefs. As hinted in the discussion of the original submission, the results change dramatically if subjects have biased priors, as there is reason to be the case in these experiments. We now allow for biased priors, and fit the degree of this bias to the data. We find that the large qualitative deviations between the optimal model predictions are largely eliminated.

We have made changes throughout the paper to address this point.

2. A somewhat related point (i.e., also discussed in Tauber et al., 2017) is the question of individual behaviour versus aggregate behaviour. The approach of the authors of fitting a single parameter set across two experiments and showing that the model simultaneously predicts both data sets is pretty impressive. However, the downside of this is that all evaluations of the model also happens on the aggregate level and are prone to the ecological fallacy. There is no indication whether any of the data pattern against which the model is compared is a pattern that holds

across individuals, for a group of individuals, or is potentially an aggregation artefact. I do not think this disqualifies the current model but this issue clearly restricts its explanatory power. I feel like that the two approaches to handle it are either providing some indication for each data pattern how strongly it holds across participants (e.g., graphically if possible) or acknowledge this limitation explicitly.

Thank you for raising this important point. Based on the reviews, we performed individual-level fitting and we present the results in the Supplementary Information. Although many of the effects are very noisy with the smaller sample size, we see that for all the plots, the qualitative pattern is shown by most participants.

3. One of the key take-home messages of the paper that the authors highlight themselves is that "the results show that the fixation process during simple choice is influenced dynamically by the value estimates computed during the decision process" (quote from the abstract). It is clear that this is an important insight going forward. However, I wonder how much of this take away is a consequence of the model. In contrast, the discussion highlights that this is mainly a behavioral pattern (see ll. 339 to 341) that needs to be explained. The current model does so, but it is unclear if it really is the only one that does so. I think it would be helpful to be clearer in how much of this insight is data driven and how much is model driven.

You raise an interesting meta-scientific question: What is the role of a model in generating insight? Clearly, the data alone strongly support an interactive and dynamic fixation process. However, much of the evidence we present is the direct result of thinking about the mechanisms and predictions of the model, in particular the patterns shown in Figures 4 and 5.

Furthermore, the model allows us to show that the *manner* in which the fixation process is dynamically influenced by the value estimates is, to a large extent, quantitatively consistent with optimal information sampling. This conclusion would be very difficult to draw without a fully implemented computational model.

4. The main text states (p. 7, 164): "We use maximum likelihood to fit these parameters to choice and fixation data." I think this sentence is an overly generous summary of the actual fitting process. It is clear that this model and the fitting process have a lot of moving parts and fitting this model requires a lot of choices. One obvious reason for this is that the complexity of the model is such that only a stochastic approach for fitting the model is computationally feasible. However, instead of using a more established approach for fitting such models (e.g., ABC, Palestro et al., 2019; likelihood-free methods, e.g., van Opheusden et al., 2020) the authors develop their own method for fitting such data which involves steps of data transformation and binning and a grid-search to find plausible parameter regions. The authors provide some assurances that this method is valid, especially for a simplified case, but we mostly have to take the authors word that they implemented everything correctly and none of the somewhat

arbitrary choices plays a too important role. Of course, the biggest argument for the authors case is the overall good performance of the model on the 50% hold-out data. To sum this up, I think one could argue if the maximum likelihood for this model and data actually exists (e.g., what is the conditional distribution of the fixation times?). To me it looks as if the authors have found a set of representative parameter estimates that produce the main qualitative patterns in the data. Maybe one could make a case that the procedure "approximates" the likelihood or "pseudo-likelihood" but even this seems a bit of a stretch.

First, we agree that our characterization of the method did not properly convey the approximate nature of the approach. We have added qualifying language in multiple places, always referring to the approach as “approximate maximum likelihood. Second, we provide additional support for our fitting approach with a parameter recovery exercise, reported in the SI. We expand on the motivation for our approach below.

We realize that our fitting method may seem somewhat arbitrary; however, we feel that it strikes a good balance between rigor and practicality, given the unique challenges involved in fitting this model (computationally intensive simulations coupled with high dimensional observations). In terms of how we approximate the likelihood, we use an approach that is inspired by ABC methods, where it is common to collapse a high dimensional observation into summary statistics. Although we could in principle estimate a kernel density estimate over these values (as in probabilistic density approximation; Turner & Sederberg 2014), the combination of several discrete and continuous dimensions makes this method much less robust than in the cases where this method has been successfully applied (fitting only choice and reaction times). Note that inverse-binomial sampling (van Opheusden et al. 2020) cannot be applied in our case because it involves sampling from the model until it produces a simulated trial that matches an observed trial. Because a trial includes the fixation sequence (which, when discretized, results in a binary/trinary sequences of around 10-50 elements, i.e. a random variable with 3^50 possible values), the chance of producing an exact match is infinitesimal. This reasoning was confirmed by personal communication with the first author of that paper.

In terms of our decision to use maximum likelihood estimation rather than MCMC sampling (as is typically used in ABC), this is primarily due to the computational cost of evaluating the likelihood (around two hours for a single parameter configuration). MCMC methods typically require a large number of likelihood evaluations, which must occur (primarily) in sequence, rather than in parallel. In contrast, we were able to achieve fairly reliable maximum likelihood estimates by evaluating the likelihood for 10,000 points in a massively parallelized architecture. We feel that the much greater cost (in terms of both time and computational resource consumption) of Bayesian methods outweighs the benefits in our specific case, especially given that we are not interested in evaluating the parameter estimates themselves.

5. I had problems understanding the "Uncertainty-directed attention" section of the paper. In particular, I did not understand what the actual DV is here. For example, why is a negative "relative cumulative fixation time at the beginning of a new fixation, starting with the second fixation" an indication for "a tendency to fixate on items that have been fixated relatively less in the trial"? Again, this might be more of a problem that I am missing the relevant background, but this was the only section of the paper in which I did not understand the measures and how they were derived from the data. Maybe this can be clarified.

Thank you for identifying this potential source of confusion! This paragraph now reads:

> Fig. 5A depicts the distribution of relative cumulative fixation time at the beginning of a new fixation, starting with the second fixation. That is, at the onset of each fixation, we ask how much time has already been spent fixating the newly fixated item, compared to the other items. In both cases, the actual and predicted distributions are centered below zero, so that items tend to be fixated when they have received less fixation time than the other items. Additionally, the model correctly predicts the lower mode and fatter left tail in the two-item case.

6. The method section contains a section on the "Optimal policy for model with random fixations". However, this policy does not any more seem to be part of the paper. Maybe it was part of a previous version?

Well this is embarrassing! Indeed, a previous version included a model comparison with this lesioned model. We removed this analysis due to concerns about the interpretation of such results with an approximate likelihood function.

7. Again, this probably shows my lack of relevant background knowledge, but it may be helpful to add an explanation of what the difference between a metalevel MDP and a regular MDP for the actual fitting is. Does it make a difference if I simulate from a regular MDP or a metalevel MDP? If so, how?

Formally, the two are identical. We use the term "metalevel MDP" as a tribute to the work in rational metareasoning that inspires our methodology. We have added a sentence to clarify this point:

> However, formally, it is identical to an MDP, and can be interpreted as such.

8. It is unclear if epsilon, the mixture weight between the approximate likelihood and the uniform error process, is also a free parameter. From the description it it seems like it, then it would be more fair to say the model as five free parameters. Also, how big is the estimate of epsilon?

We do not treat epsilon as a free parameter because it is not part of the simulation model (and hence does not contribute to the model's ability to replicate the patterns illustrated in the figures). Epsilon only contributes to the likelihood approximation. Thus, we view epsilon as a heuristic technique to address the computational challenges associated with fitting a complex model to high dimensional data (we cannot possibly simulate enough trials to provide complete coverage of the high dimensional histogram). We have added the following to the methods to clarify this point:

> Importantly, this error model is only used to approximate the likelihood; it is not used for generating the model predictions in the figures—indeed, it could not be used in this way because the error model is defined over the summary statistics, and cannot generate full sequences of fixations. Thus, the ε parameter should be interpreted in roughly the same way as the bandwidth parameter of a kernel density estimate [87], rather than as an additional free parameter of the model.

For poorly fitting parameters, epsilon takes the maximum value of 0.5. For the best fitting parameters which we show in the plots, epsilon was (mean ± SD) 0.124 ± 0.015.

9. The method section states "thus, to give a rough sense of the uncertainty in the estimate, we identify the top thirty parameters, giving us both mean and standard error estimates for each parameter and the total likelihood." The main text gives the parameter estimates as "mean ± std". Please clarify: Is the "std" in the main text the standard error of the parameter estimates (i.e., SD of the 30 top estimates divided by sqrt(30)) or simply the SD of the 30 top estimates? If the former I am not sure I understand how it makes sense to divide by sqrt(30) here. The 30 seems to be an arbitrary cut-off point (to denote something like "close enough") but this would then not justify to use this as the sample size in the standard error calculation.

We apologize for this error. We have corrected the methods to read "giving us both mean and *standard deviation*". We have also replaced "std" with "SD".

10. The method section states "Based on manual inspection, we identified the promising region of parameter space to be ... A grid search confirmed that this area was likely to contain the global optimum (see Fig. S4)." However, with the exception of one parameter, y_switch, figure S4 only shows the range given in this part of the text. Thus, it does not permit the inference that the chosen region is the promising region as essentially no information about values outside the region are available.

Excellent point. Given this concern (and the monetary cost of producing the plot), we elected not to regenerate it with the additional prior bias parameter, α. We have thus removed the sentence and the plot.

Minor issues:

- l. 81: "during simple is influenced". Seems like "decision making" is missing here after "simple".

Thanks, fixed!

- Figure 2, note: "This probability correlates strongly with the value of sampling the item". I wonder why it says "correlates strongly". What is missing from a perfect correlation (only the switching costs)?

The full value of sampling depends on three additional factors: (1) the size of the difference in value from the current leading item matters as well because changing your decision is more valuable if you determine that the ultimately chosen item is much better than the previous leader as opposed to just slightly better, (2) the cost of sampling necessary to change one's mind (this may include switching costs), and (3) the possibility of deciding to sample another item, e.g. the current leading item, at a future point. We have added the following parenthetical to clarify this point

> (the full value of sampling additionally depends on the size of the potential gain in value, as well as the cost of future samples and the possibility of sampling other items)

- l. 512: "liking ratings provided by the participants." seems to be wrong here.

Thanks, fixed!

- l. 547: "between" appears twice.

Thanks, fixed!

Responses to R3

We thank the reviewer for the careful reading of the paper and the useful comments. As you will see, we have carried out a substantial additional number of analyses that we hope have further strengthened the paper.

For the reviewer's convenience, we have copied below the original comments in regular font-type, and added our responses in bold. Note also that a draft with inline figures (and matching line numbers) is available here:
http://fredcallaway.com/pdfs/callaway-plos-attention.pdf

COMMENTS AND RESPONSES

It has been a pleasure to read the paper from Callaway and colleagues on fixation patterns in value-based choice and optimal information sampling. I think this is a very timely paper. In the last decade many studies have investigated the role of attention in value-based choice using models like the aDDM, LCA or the model from Cavanagh et al. However, a shortcoming/oddity of these models is that attention is treated as an exogenous variable and therefore does not provide a satisfactory explanation of where the attention pattern originated. This work attempts to fill in this gap by proposing a generative model of attention based on optimal information sampling, studying its effect on value based choice in binary and trinary decisions. The manuscript is well written and the authors present the results of their data fitting to previous studies in a straightforward way. I particularly appreciated the fact that the authors point out to the reader the cases where their model undershoots, overshoots, or completely fails to predict some of the data. Below are a few suggestions that I hope the authors will find useful in their revisions:

Thank you for the encouraging and helpful comments!

1) It looks like that their model works at its best for 3 options setting (but note that also in this case it is often off mark - e.g. fig 6.B). In fact, while the model captures some of the basic behavioural tendencies for the 2 options task, it completely fails to predict some of the key effects for which the aDDM was developed in the first place (i.e. value-directed attention and choice bias). I appreciate the approach of developing a model with minimal assumptions. But these failures in the 2-choice setting are not just small wrinkles for a model of attention as optimal information sampling. While the authors acknowledge this shortcoming, they don't provide a compelling explanation of what causes this and how this can be fixed. They mention in the discussion another approach (used in another recent study) that uses a negatively biased prior. But they say in the supplemental that they tried a similar approach "was not sufficient to even qualitatively capture the bias in the binary choice". However, eyeballing the figure S1B compared with 7B it looks that some of the shape in the data is captured by using biased priors.

Is using biased prior the only way to fix this problem or the authors have other intuitions that can help for the binary case? In any case this issue needs to be investigated in more details. Unfortunately the behavioural effect of choice bias and value-directed attention are robust findings at the core of many previous papers using aDDM. I therefore think that this shortcoming cannot be easily swept under the carpet. Models with minimal assumption are fine, but not if they fail to capture (even qualitatively) such strong effects. My fears are double here: 1) such mismatch between data and model might be a clear indication that the authors are missing some key ingredients in their assumptions 2) that the paper will not be not so impactful (or even worst dismissed) by people in the field of DM since most studies use binary choice. Since a lot of good work has gone into this, that would be a real shame. Therefore, I suggest putting more work into understanding what causes these mismatch and how think how it can be ameliorated (even if this comes at a cost of increasing model complexity).

Thank you very much for this invaluable suggestion! It turns out that our initially unpromising results in fitting the prior were due to not investing the necessary computational resources to fully explore the parameter space when performing model fitting. Inspired by your comment, we invested said resources and we now report results with the prior mean fit to behavior. The model now (qualitatively) captures all the choice bias effects in both binary and trinary choice, and we have added a substantial amount of material to the paper discussing the important role the prior plays in producing this effect.

2) For the 3 options experiment most analyses are based on a specific definition of DV X - mean of other 2 options. While this approach has been used in some previous papers, I find it a bit arbitrary. Who decides that participants are evaluating that item against the mean of the other 2 is the right operation? why not the median or a weighted average? or is just considering 2nd best ignoring completely the worst item?. I suggest a more agnostic approach, using multinomial logistic regression model in which the value of each option is inputted independently and not requiring a priori specification of DV.

We completely agree that the trinary choice data could be analyzed using multinomial models, instead of the "reduced logistic approach" that we use in our plots . We also agree that the "reduced logistic graphs" that we show could be based on a number of statics, and that the ones that we use, although previously used in the literature, are not the only options.

Note that if the models match the multinomial structure of the data well, this would also show up in the 'reduced logistic graphs' that we use. Note also that there is no post-hoc bias in which 'reduced logistic' representations we show: the choice was simply guided by the previous literature.

Given the already substantial complexity of the paper, the fact that it is not clear how to use multinomial logistic regression for producing graphical representations, and that our goal is simply to test a large number of sophisticated and quantitative model predictions, we have a strong preference to leave these

analysis as they are. Needless to say, however, we would be happy to carry them out if the reviewer views this as critical.

3) Sum value (i.e. the value of both option) has been used in a number of studies including some recent work from Krajbich group. This has enabled the authors to distinguish between different competing dynamic models (in particular arbitrating between additive vs multiplicative effect of attention). I am wondering if is worth investigating what their optimal model predict for sum value effect on attention. That might give some interesting new insights.

Excellent point! Note that R1 had a similar comment. We copy our previous discussion of the overall value effect here for the reviewer's convenience:

We now include the overall value effect in Figure 3. Our model predicts a strong overall value effect because Bayesian updating implies a multiplicative effect of attention (as does the aDDM). In particular, the expected estimated value after N samples of an item is:

$$E[\mu] = \mu_0 + [(N * \sigma_2^{-2}) / (\lambda_0 + N * \sigma_2^{-2})] * (true\_value - \mu_0)$$

That is, the expected prior mean is a hyperbolic function of the amount of attention with start and end point determined by the prior mean and the true value (Armel & Rangel 2008).

We also briefly discuss how something like an additive effect can emerge in our model as a result of value-directed attention (in the Discussion).

> Our results also suggest another (not mutually exclusive) mechanism by which the attention- choice correlation can emerge: value-directed attention. We found that the optimal model with no prior bias (α = 1) predicts an attention-choice correlation in the trinary choice case. This is because, controlling for true values, an increase in estimated value (e.g., due to sample noise) makes the model more likely to both fixate and choose an item. This could potentially help to resolve the debate over additive vs. multiplicative effects of attention on choice [11, 13]. While the prior-bias mechanism predicts a multiplicative effect, the value-directed attention mechanism predicts that fixation time and choice will be directly related (as predicted by the additive model). Although we did not see strong evidence for value-directed attention in the binary dataset, such a bias has been shown in explicit information gathering settings [65] and could be at work in other binary choice settings.

4) The work by Hébert and Woodford 2019 seems relevant here but is not currently discussed.

We apologize for this omission. This is a very interesting point of comparison indeed! We have added the following paragraph to the Discussion:

> Our work most closely relates to two recent lines of work on optimal information sampling for simple choice. First, H ébert and Woodford [66, 67] consider sequential sampling models based on rational inattention. They derive optimal sampling strategies under highly general information- theoretic constraints, and establish several interesting properties of optimal sampling, such as the conditions under which the evidence accumulation will resemble a jump or a diffusion process. In their framework, the decision maker chooses, at each time point, an arbitrary information struc- ture, the probability of producing each possible signal under different true states of the world. In contrast, we specify a very small set of information structures, each of which corresponds to sampling a noisy estimate of one item's value (Equation 1). This naturally associates each infor- mation structure with fixating on one of the items, allowing us to compare model predictions to human fixation patterns. Whether human attention more closely resembles flexible construction of dynamic information structures, or selection from a small set of fixed information structures is an interesting question for future research.
