# Supplementary material for: Fixation patterns in simple choice reflect optimal information sampling
Source: PLoS Comput Biol. 2021 Mar 26;17(3):e1008863. doi: 10.1371/journal.pcbi.1008863 (PMC8026028; doi:10.1371/journal.pcbi.1008863)
Supplement: Attachment — Submitted filename: plos-response-2.pdf [file pcbi.1008863.s004.pdf]

Dear Dr. Gershman,

Please find enclosed a revised version of our manuscript “Fixation patterns in simple choice reflect optimal information sampling” (manuscript number PCOMPBIOL-D-20-01568).

We are very pleased to hear that you and all the reviewers have found the manuscript to be improved. This improvement is largely the result of the invaluable comments provided by the reviewers. We have thus added an acknowledgement of the essential role the reviewers played in the creation of this paper:

“We thank three anonymous reviewers for very useful comments during the review process.”

Summary of revision changes

- We have corrected the typos identified by R2 and also several additional minor typos and language errors identified by an automatic proofreading tool.
- We have added four references (listed below) to related work.
- We have made several small changes to the text to minimize the possibilities for confusion suggested by R1.

We provide a more detailed response to R1’s comments on the following page

Best,

Fred Callaway, Antonio Rangel, Tom Griffiths

Response to R1

Thank you for the continued very useful comments, and apologies for misunderstanding some of your earlier comments. We are hopeful that we understand these nuanced points better now and that we have addressed it adequately in the text and below.

1. I would like to clarify the issue with the term "sequential". What I meant is that both the DDM and aDDM are sequential sampling models in which evidence is sampled (sequentially) in a relative fashion, so it is always evidence for A relative to B that it sampled (as in the Sequential probability ratio test). This is what distinguishes sequential models in which evidence is accumulated "in a single sum" (such as DDM, aDDM, DFT, OU models) from sequential sampling models in which evidence is accumulated in separate accumulators (such as race models, LCA, etc). Therefore, I would like to stress that when the authors were using the term "sequential" to refer to the fact that evidence was accumulated sequentially for different options, this does not correspond to neither what the DDM or the aDDM "do".

Therefore, I suggest to change the term "sequential" with perhaps "time-varying accumulation rates" or something similar. I think it should be clear that the authors are not suggesting that when looking at A you are not accumulating evidence for B (again, the accumulated evidence is relative for A vs. B, not absolute for A in both the DDM and aDDM). However, the crucial addition of the aDDM vs. the DDM is that it allows the accumulation rate to vary within the trial based on attentional shifts.

This is a valid point. We certainly did not intend to suggest "that when looking at A you are not accumulating evidence for B" and we agree that it is critical that we prevent the possibility that the reader infers such a suggestion. By "sequential" we only mean that multiple value samples are taken over a period of time. We have ensured that we never use the term "sequence" or "sequential" in a context where it could be interpreted in this way. This involved removing one occurrence (l. 82):

> In contrast, the optimal algorithm when the decision-maker must sample information ~~sequentially and~~ selectively is unknown

We have also added the following clarification to the introduction (l. 28-29):

> These findings have been explained by the Attentional Drift Diffusion Model (aDDM), in which the value samples of the fixated item are over-weighted relative to those of unfixated ones **(or equivalently in the binary case, discounting the influence of the unattended item on the drift rate)**

2. The other clarification is about inhibition. I am not sure what the authors mean by "In the traditional two-alternative choice DDM (and the original aDDM) the role of inhibition depends

on the exact neural network used to implement or approximate the algorithm". In Bogacz 2006 (The Physics of Optimal Decision Making: A Formal Analysis of Models of Performance in Two-Alternative Forced-Choice Tasks) it is shown how the DDM relates to other sequential sampling models only when inhibition is high or there is perfect inhibition.

Moreover, since the DDM is related to the SPRT, I am not sure I get why the author say that "Adding inhibition would necessarily violate the rational norm of Bayesian inference, and thus does not seem appropriate for our model given our emphasis on optimality". For what I understand, mutual inhibition is necessary for optimality in sequential sampling models. Perhaps the authors can elaborate/better explain their point.

We are sorry that these comments regarding inhibition in our previous response letter were not as clear as they should have been. Fortunately the confusion does not seem to have leaked into the paper.

Here are some thoughts on this.

First, we actually had the Bogacz paper in mind when writing our previous response. That paper beautifully shows how various accumulator models with inhibition are analogous to the DDM, under appropriate combinations of parameters.

Second, one can also devise multi-unit accumulators that are equivalent to the DDM, *without inhibition*. This is what we meant by "the role of inhibition depends on the exact neural network used to implement or approximate the algorithm".

In particular, some previous specifications of the DDM do not involve any explicit inhibition. For example, in the binary DDM, there is only one accumulator and thus there is no place for inhibition. We can also express the DDM with two accumulators and a relative decision value defined as the difference between the values of the two accumulators.

$$E_{t+1}^{(1)} = E_t^{(1)} + dr^{(1)} + \varepsilon_t^{(1)}$$

$$E_{t+1}^{(2)} = E_t^{(2)} + dr^{(2)} + \varepsilon_t^{(2)}$$

$$V_t = E_t^{(1)} - E_t^{(2)}$$

$$\implies V_{t+1} = V_t + d\left(r^{(1)} - r^{(2)}\right) + \varepsilon_t$$

Note that the joint noise term ε_t has twice the variance of the individual noise terms. We see that the two-accumulator model reduces to the DDM without introducing any inhibition between the accumulators. However, one could very reasonably argue that the subtraction in defining the relative value signal is a form of inhibition. In that sense, our model also has inhibition because the decision to stop sampling is based on differences in estimated values. We were not considering this comparison to fall under "inhibition" in our original response.

Third, we view inhibition as a process-level mechanism in which neural populations directly inhibit each other. Such a mechanism may be critical in a biologically plausible implementation/approximation of our model; however, our more abstract model is silent about these issues.

Finally, although these issues were related to our previous response letter, and not the paper, inspired by the reviewer's comment we have added the following new final sentences of the discussion (l. 608-613):

> Finally, in contrast to many sequential sampling models, our model is not intended as a biologically plausible process model of how the brain actually makes decisions. Exploring how the brain might approximate the optimal sampling policy presented here, and also how optimal sampling might change under accumulation mechanisms such as decay and inhibition is another priority for future work.

Added references

19. Thomas AW, Molter F, Krajbich I, Heekeren HR, Mohr PNC. Gaze Bias Differences Capture Individual Choice Behaviour. Nature Human Behaviour. 2019;3(6):625–635.

22. Sepulveda P, Usher M, Davies N, Benson AA, Ortoleva P, De Martino B. Visual Attention Modulates the Integration of Goal-Relevant Evidence and Not Value. eLife. 2020;9:e60705.

23. Moreno-Bote R, Ram´ırez-Ruiz J, Drugowitsch J, Hayden BY. Heuristics and Optimal Solutions to the Breadth–Depth Dilemma. Proceedings of the National Academy of Sciences. 2020;117(33):19799–19808.

24. Ram´ırez-Ruiz J, Moreno-Bote R. Optimal Allocation of Finite Sampling Capacity in Accumulator Models of Multi-Alternative Decision Making. arXiv:210201597 [q-bio]. 2021;.
